# Understanding the within-host dynamics of influenza A virus: from theory to clinical implications

**DOI:** 10.1098/rsif.2016.0289

**Published:** 2016-06

**Authors:** Christoforos Hadjichrysanthou, Emilie Cauët, Emma Lawrence, Carolin Vegvari, Frank de Wolf, Roy M. Anderson

**Affiliations:** 1 Department of Infectious Disease Epidemiology, School of Public Health, Imperial College London, London, UK; 2 Janssen Prevention Center, Leiden, The Netherlands

**Keywords:** viral kinetics, immune control, acute viral infection, within-host modelling, influenza A

## Abstract

Mathematical models have provided important insights into acute viral dynamics within individual patients. In this paper, we study the simplest target cell-limited models to investigate the within-host dynamics of influenza A virus infection in humans. Despite the biological simplicity of the models, we show how these can be used to understand the severity of the infection and the key attributes of possible immunotherapy and antiviral drugs for the treatment of infection at different times post infection. Through an analytic approach, we derive and estimate simple summary biological quantities that can provide novel insights into the infection dynamics and the definition of clinical endpoints. We focus on nine quantities, including the area under the viral load curve, peak viral load, the time to peak viral load and the level of cell death due to infection. Using Markov chain Monte Carlo methods, we fitted the models to data collected from 12 untreated volunteers who participated in two clinical studies that tested the antiviral drugs oseltamivir and zanamivir. Based on the results, we also discuss various difficulties in deriving precise estimates of the parameters, even in the very simple models considered, when experimental data are limited to viral load measures and/or there is a limited number of viral load measurements post infection.

## 1. Introduction

Influenza continues to be a significant cause of morbidity and mortality worldwide [1]. Seasonal epidemics of influenza cause more than 300 000 deaths annually around the world. In the USA alone, a typical seasonal influenza A epidemic results in over 200 000 hospitalizations [2] and 36 000 deaths [3].

Influenza is a short-lived infection with an incubation period of approximately 2 days [4]. The standard pattern of virus kinetics is characterized by rapid exponential growth, with a peak in viral load occurring 1–3 days post infection, followed by a decline over the subsequent 3–5 days. In patients with immunodeficiency, the duration of infection may be prolonged [5,6].

Experimental studies on the typical course of influenza A in a patient have provided useful insights into the processes controlling viral dynamics, especially the associated immune response. Mathematical models have been used to improve understanding of the infection dynamics. Influenza A virus kinetics in the human body has been examined in a number of previous studies [7–10]. Various models that describe the infection dynamics in animals (for example in mice [10–12] and horses [13,14]) have also facilitated the investigation of the immunological mechanisms involved in controlling influenza A replication. Models have been developed to incorporate the innate immune response, the adaptive response or both types of responses against influenza A [7–9,11–18]. Mathematical modelling has also helped in assessing the efficacy of influenza antiviral treatments [19–22] such as neuraminidase inhibitors (oseltamivir and zanamivir) [7,8,17,23,24] and adamantanes (amantadine and rimantadine) [17,25]. The models developed typically consist of systems of ordinary differential equations (ODEs), based on the classic viral dynamic model describing uninfected and infected cells and free virus in the host [7,26–31]. Stochastic effects become important when the viral load is at low levels [8] (for reviews of the mathematical model development, see [32–34]).

Despite the availability of a range of mathematical and statistical tools, limitations in data availability and measurement issues in clinical studies of infected patients hinder the development of complex models that will accurately predict the infection dynamics and provide insight into the exact mechanisms responsible for its control. In this paper, we consider the simplest within-host models of viral dynamics. We assess the validity of these models by fitting to data collected from 12 untreated volunteers experimentally inoculated with human influenza A, but left untreated by drugs or immunotherapeutic agents. Despite the biological simplicity of these models, they can facilitate the assessment of infection-related morbidity and the efficacies of immunotherapies or antiviral treatments and help in the design of control and prevention strategies. We derive and estimate simple quantities that reflect the severity of the infection at different times post acquisition and can be considered as potential endpoints in clinical trials. Such quantities include the area under the viral load curve, the peak viral load, the time to peak viral load and the level of cell death due to infection. The analytical results derived shed light on important parameters that influence patterns in within-patient viral kinetics. Based on the results, we also discuss problems arising in predicting the viral dynamics and evaluating therapeutic interventions when the quality of clinical data on viral load is suboptimal. We provide guidance on what to measure, and when and how frequently, in order to accurately describe the infection dynamics and facilitate the accurate assessment of therapies that restrict viral growth.

## 2. Material and methods

### 2.1. Simple mathematical models of viral dynamics

The main effects of the immune response on viral populations can be simply classified as one of the following: (i) decreasing the infection of susceptible cells, (ii) reducing the production of virus by infected cells, (iii) killing infected cells, and (iv) increasing the clearance rate of free virus particles. The simplest model of influenza A virus infection that incorporates implicitly or explicitly these four mechanisms is the TIV model [7]. This model assumes that susceptible cells (primarily epithelial cells), *T*, become infected when in contact with free infectious virus particles, *V*, via a mass-action process with rate *β*. Virus is produced in direct proportion to the number of (productively) infected cells *I* at rate *p* per infected cell and is lost at rate *c* per virion due to non-specific mechanisms that include immune response and natural virus decomposition. An infected cell dies or is killed by immune cells and other non-specific mechanisms with rate *δ_I_*. Hence, infected cells produce an average *p*/*δ_I_* virions during their lifetime. The ODEs that describe this dynamical system are as follows:2.1
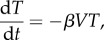
2.2
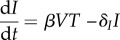
2.3
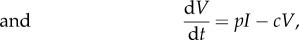
with initial conditions2.4
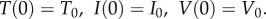


**Figure 1.**
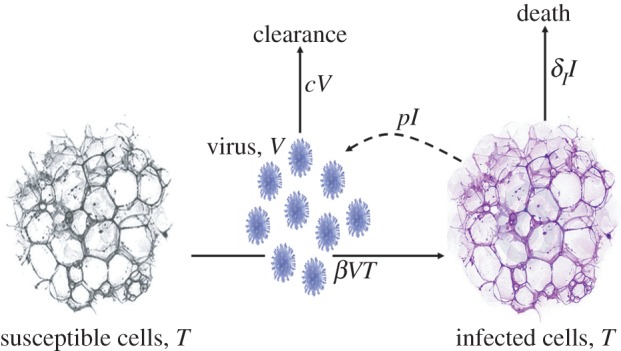
A schematic diagram of the TIV model (2.1)–(2.4) of viral dynamics. (Online version in colour.)

The TIV model, (2.1)–(2.4), is shown schematically (figure 1).

Owing to the paucity of quantitative information on the clearance rate of the virus and the death rate of infected cells, we focus on a simpler version of the TIV model which assumes that the viral dynamics is much faster than the infected cell dynamics and that a quasi-stationary state at which *V* = *pI*/*c* is attained very quickly [35–37]. In this case, the TIV model is reduced to the following pair of equations:2.5
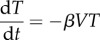
and2.6
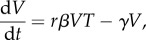
with initial conditions2.7
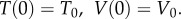


The parameter *γ* is the death rate of infected cells *δ_I_* in the TIV model and *r* can be interpreted as *p*/*c*. However, as the parameters *p* and *c* cannot be estimated independently, we will consider *r* as a single parameter. Model (2.5)–(2.7) will be referred to as the TV model. The TV model has the same structure as the susceptible–infectious–recovered (SIR) model in infectious disease epidemiology of viral spread in a population of hosts [38–40] and thus most of the results derived for the SIR model in host populations can be applied in the context of within-host viral dynamics.

The TV model can be extended to the so-called TVA model to include a representation of the overall action strength of the immune response against influenza A (see electronic supplementary material, S1). All three models, TIV, TV and TVA, predict changes in viral load over time post infection accurately. However, the TV and TVA models are ‘better’ models; the fit of the TIV model to observed viral load data is almost equivalent to that of the TV and TVA models (data are not shown) and there are no data to support the estimation of the extra parameters in the more complex model. As there is no independent information about the dynamics of infected cells, the quasi-stationary state assumption in the TV and TVA models is therefore reasonable. We focus on the TV model, but in the electronic supplementary material, S3, we add to previous published analytical results for the TIV model and in the electronic supplementary material, S1, we discuss the TVA model in more detail.

Table 1 summarizes the variables and parameters of all the models considered in this paper.

**Table 1.** Notation of the models' variables and parameters.

notation	meaning	units
model variables		
*T*	cells susceptible to infection	cell
*I*	infected cells	cell
*V*	viral load	TCID_50_ ml^−1^
*A*	immune response	
model parameters		
*β*	infection rate	(TCID_50_/ml)^−1^ × day^−1^
*p*	viral production rate per infected cell	TCID_50_ ml^−1^ × day^−1^
*c*	viral clearance rate	day^−1^
*δ_I_*	death rate of an infected cell	day^−1^
*γ*	viral decay rate	day^−1^
*r*	rate at which target cells that become infected produce virus in the TV and TVA models	TCID_50_ ml^−1^
*w*	clearance rate of virus by immune response	day^−1^

The above models encapsulate a number of biological assumptions. For example, the regeneration and natural death of target cells have been neglected due to the long time scales of these processes compared with the time scale of influenza infection [41,42]. Therefore, even in cases of severe infection the virus will eventually decline due to the depletion of susceptible target cells. Including the regeneration of target cells does not improve the model fit in most cases [7,10–14,25,43]. An additional assumption is that there is no delay between cell infection and production of virus (a latent phase). Moreover, it should be noted that if no death of infected cells occurs during the latent phase, then the delay simply postpones the infection dynamics (delays the onset of the infection, reduces the peak of the viral load and increases the duration of the infection) without reducing the amount of viral shedding significantly (if regrowth of susceptible cells is considered then increasing the delay of virus production will result in a reduction in the amount of viral shedding). Finally, the loss of virions through cell entry is also considered negligible and absorbed into the loss term −*cV*, given that any one cell has the potential to produce between 10^3^ and 10^4^ virions [44], which is much more than the number of virions needed to infect a cell [45]. This loss might be important in *in vitro* models [25].

### 2.2. Parameter estimation

#### 2.2.1. Datasets used

We use viral load data from two different datasets: the first consists of six volunteers from the placebo group of the oseltamivir trial conducted by Roche [46]. All the participants were healthy adults and screened for haemagglutination inhibition titre. Intranasal inoculation with 10^6^ (50% tissue culture) of a safety-tested pool (TCID_50_) of human A/Texas/36/91 H1N1 influenza virus was performed on day 0. Nasal lavage fluids were collected for virus isolation and titration by standard methods on days 2–8. The second dataset also consists of six volunteers who were part of the placebo group of the zanamivir trial conducted by GlaxoSmithKline (GSK). The volunteers in this trial were also inoculated with human H1N1 influenza virus (A/Texas/91 H1N1) following a similar procedure.

For the model fitting and the estimation of the parameters, when two or more (sequential) viral load data points fall below the detection limit we exclude them all but the first one. In this case, the first undetectable viral load data point is set to be equal to the value of the detection limit. In the data analysed in this study, the value of the detection limit was not known and it was set to be equal to 0.7 TCID_50_ ml^−1^, which is 0.05 TCID_50_ ml^−1^ below the smallest measurement value in the two datasets considered.

#### 2.2.2. Fitting procedure

In the TV model the parameters *r*, *I*_0_ and *T*_0_ cannot be estimated independently. We fixed *T*_0_ at 4 × 10^8^ cells [7] and *I*_0_ at 0. A structural identifiability analysis of the TV model [47] shows that the estimation of the identifiable parameters (*β*, *γ*, *r* or *T*_0_) requires at least five measurements of viral load at distinct time points. However, examining the pairwise relationships between the parameters (see §3.2), it is observed that all the parameters are correlated and it is thus not possible to produce good estimates of each individually. Owing to the correlation between *β* and *r*, we reparametrized the model by replacing *rβ* in equation (2.6) by *l* and we estimated *l* and *β* (note: *β* and *l* are also correlated).

We fitted the TV model to data and estimated the parameters for each patient independently using a random-walk Metropolis–Hastings algorithm. Independent prior distributions were chosen for each of the unknown parameters *V*_0_, *β*, *l* and *γ*. An exponential distribution with mean 100 was chosen for *V*_0_ and *γ*. A uniform distribution [0–0.003] (except for patients 3 and 4 in the Roche -dataset, [0–0.01]) was chosen for *β* while a uniform distribution [0–0.0000001] was chosen for *l*. It was assumed that the measured viral loads were lognormally distributed around the true viral loads [48] with standard deviation 0.3 log_10_ (this was based on data provided in [49], but preliminary studies show that the assumed standard deviation of the lognormal distribution does not significantly affect the parameter estimates). For each individual dataset, 8.1 × 10^5^ sampling iterations were performed, of which the first 10^4^ iterations were discarded. To reduce autocorrelation, every 500th sample was recorded [50]. Convergence was assessed visually from the traces of each parameter.

### 2.3. Infection-related quantities

**Figure 2.**
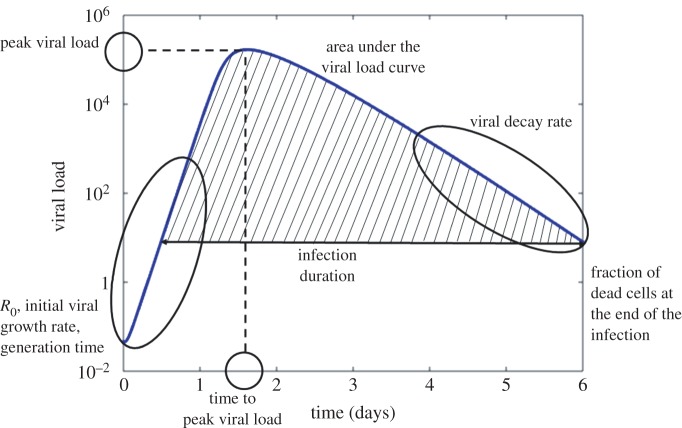
Summary of the infection-related quantities: the basic reproduction ratio, the initial viral growth rate, the generation time, the peak viral load, the time to peak viral load, the infection duration (time interval in which the viral load is above a threshold), the area under the viral load curve above a threshold and the fraction of dead cells. (Online version in colour.)

We derive either exact or approximate solutions of nine infection-related quantities that can help us to interpret the viral load scores in terms of infection severity at both the individual and the population level. The quantities considered are the following: the basic reproduction ratio *R*_0_ and the viral growth rate *r*_0_, which indicate the speed at which the virus disseminates within the human host at the early stage of infection; the generation time *T*_g_ [51], which indicates the speed at which the infection spreads in the population; the area under the viral load curve *A*_V_ [9,33], which indicates the infectiousness of the host; the peak viral load *P*_V_ [33], which is thought to be correlated with symptom scores; the time to peak viral load 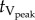 [10,17], which can be used for assessing infection progression; the viral decay rate *r*_d_, which is the rate of viral clearance at the late stage of infection; the duration of infection *t*_d_ [33], which shows the time scale of the infection and can indicate the time in which the patient is infectious; and the fraction of dead cells at the end of the infection *D* [10,33], which indicates the damage to the tissue caused by the infection, the occurrence of some respiratory symptoms and the time to recovery. The quantities are summarized in the schematic in figure 2. For more information about these quantities and the importance of each of them, see electronic supplementary material, S2.

## 3. Results

**Figure 3.**
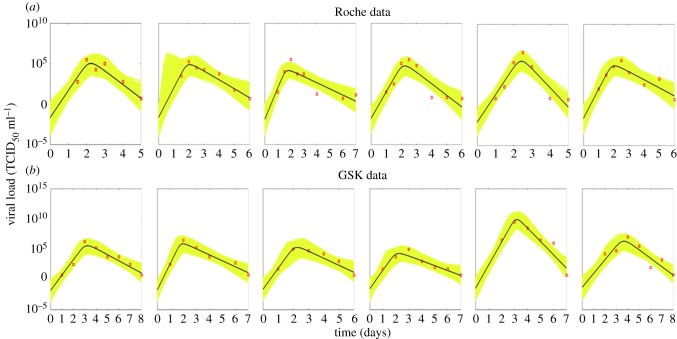
TV model fit: the black line represents the median estimate of viral dynamics (in log_10_ scale) and yellow lines are viral dynamic curves based on 10 000 samples from the posterior distribution of the parameters. Red squares are viral load data points. (*a*) Placebo-group patients from the oseltamivir trial (Roche dataset); (*b*) placebo-group patients from the zanamivir trial (GSK dataset).

The TV model fits to the placebo data and the uncertainty in the model solution is illustrated in figure 3. Despite the simplification of the complex biological process, the TV model provides a good description of the data for most of the subjects.

### 3.1. Infection-related quantities

**Figure 4.**
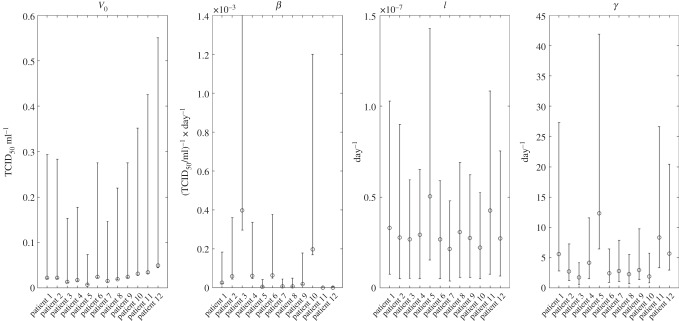
Estimated posterior medians of individual parameters and the corresponding 95% credible intervals for the 12 patients in the two datasets. The estimated values of the individual parameters are presented in the electronic supplementary material, table S5.

**Figure 5.**
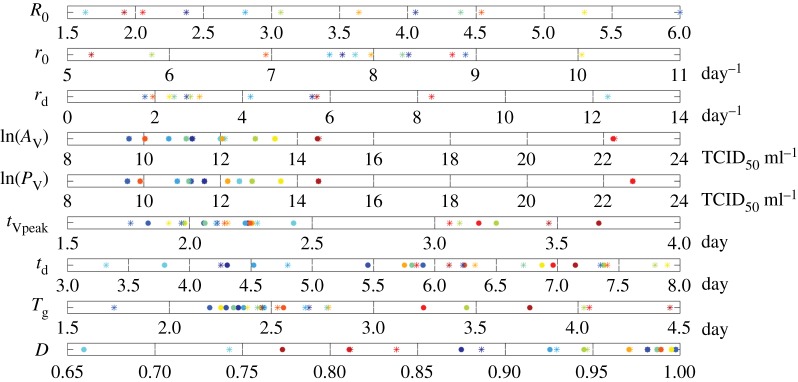
Scatter plots illustrating the variability of the infection-related quantities between the 12 patients in the two datasets. Each colour represents a patient. Numerical solutions are represented by filled circles and analytical solutions by asterisks. The estimated values of the quantities are presented in table 4.

In table 2, we summarize how the infection-related quantities in the TV model are affected by the administration of treatments that decrease the rate of infection (e.g. adamantane antiviral drugs [17,25]) and the viral production rate (e.g. neuraminidase inhibitors [7,8,17,23]) and hypothetical treatments that increase the virus clearance rate (e.g. monoclonal antibodies [52]). We also present the influence of the initial viral load. The results presented are for cases where the basic reproduction ratio *R*_0_ is greater than 1. A general conclusion is that the time of treatment during the infection is very important and, based on the models considered, its effect is highly dependent on the number of the remaining susceptible cells at this time. In table 3, we summarize the main determinants of some of the infection-related quantities as defined by the mathematical expressions derived. In table 4, we show the estimates of the infection-related quantities in the TV model based on the parameter estimates presented in the electronic supplementary material, table S5. We present both the numerical and analytical solution showing the good accuracy of the analytical results based on novel approximations. Figures 4 and 5 illustrate, respectively, the variability of the parameter estimates and the variability of the infection-related quantities between patients.

**Table 2.** Sensitivity of infection-related quantities to changes in parameter values in the TV model.

		infection-related quantities in the TV model								
		basic reproduction ratio	initial viral growth rate	late viral decay rate	area under the viral load curve	peak viral load	time to peak viral load	duration of infection	generation time	fraction of dead cells
action	decreasing *β* decreasing *r* decreasing *T*_0_	decrease	decrease	almost constant	decrease^a^	decrease	increase	increase^b^	increase	decrease
	increasing *γ*	decrease	decrease	increase	decrease	decrease	increase^c^	decrease^d^	increase^e^	decrease
	decreasing *V*_0_	constant	almost no effect	almost no effect	decrease^f^	decrease^f^^,^^g^	increase	increase^f^	increase	decrease^f^

^a^Above a threshold of *β*, all the target cells become infected and further increase of *β* does not affect *A*_V_.

^b^As *r* decreases, a decrease is observed when *R*_0_ is relatively large and all (or almost all) cells get infected.

^c^A decrease is observed when *R*_0_ is large and by the time of the peak the majority of cells die due to infection.

^d^An increase is observed in cases where *R*_0_ is relatively small and there is availability of target cells.

^e^A decrease is observed for relatively large values of *R*_0_ when the majority of the susceptible cells become infected.

^f^The variation is small.

^g^Peak viral load increases almost as much as *V*_0_ increases.

**Table 3.** Summary of the main parameters determining the infection-related quantities as derived from analytical results.

infection-related quantities	area under the viral load curve	fraction of dead cells	time to peak viral load	peak viral load	viral decay rate
mainly influenced by	*R*_0_	*R*_0_	all parameters, including *V*_0_	viral production rate (TV model)	death rate of infected cells, virus clearance rate

**Table 4.** Numerical (NS) and analytical (AS) solution of the infection-related quantities based on the posterior medians of the parameters of the TV model presented in the electronic supplementary material, table S5. The numerical solutions of the area under the viral load curve and fraction of dead cells have been compared with the simplified approximations (S3A.15) and (S3B.5) in the electronic supplementary material, S3A and S3B, respectively.

				estimates of the infection-related quantities								
				basic reproduction ratio (dimensionless)	initial viral growth rate (day^−1^)	late viral decay rate (day^−1^)	area under the viral load curve (TCID_50_ ml^−1^)	peak viral load (TCID_50_ ml^−1^)	time to peak viral load (days)	duration of infection (days)	generation time (days)	fraction of dead cells (dimensionless)
human influenza	A/Texas/36/91 H1N1 (Roche)	patient 1	NS	2.3764			7.7769 × 10^4^	1.0676 × 10^5^	2.2369	4.3051	2.3369	0.8750
			AS		7.6915	5.5881	7.8805 × 10^4^	1.0676 × 10^5^	2.1109	4.2552	2.6827	0.8866
		patient 2	NS	4.0559			6.6616 × 10^4^	7.5683 × 10^4^	2.0584	5.4530	2.2789	0.9813
			AS		8.3454	2.7309	6.6629 × 10^4^	7.5683 × 10^4^	1.9673	6.2280	2.4577	0.9815
		patient 3	NS	5.9974			1.4972 × 10^4^	1.4289 × 10^4^	1.8299	5.9046	2.1974	0.9975
			AS		8.8971	1.7803	1.4976 × 10^4^	1.4289 × 10^4^	1.7592	7.3469	1.7292	0.9975
		patient 4	NS	2.8085			4.2186 × 10^4^	5.2681 × 10^4^	2.2267	4.5206	2.3626	0.9257
			AS		7.5676	4.1844	4.2360 × 10^4^	5.2681 × 10^4^	2.1077	4.8000	2.6650	0.9295
		patient 5	NS	1.6335			1.6271 × 10^5^	2.6616 × 10^5^	2.4222	3.7942	2.4662	0.6595
			AS		7.8186	12.3417	1.8319 × 10^5^	2.6616 × 10^5^	2.2766	3.3192	2.7704	0.7425
		patient 6	NS	4.3905			6.6905 × 10^4^	7.2093 × 10^4^	2.0627	5.8110	2.3130	0.9869
			AS		8.2828	2.4429	6.6912 × 10^4^	7.2093 × 10^4^	1.9743	6.7194	2.4321	0.9869
	A/Texas/91 H1N1 (GSK)	patient 1	NS	3.0683			4.0336 × 10^5^	3.7139 × 10^5^	3.2494	7.3740	3.4535	0.9449
			AS		5.8292	2.8184	4.0419 × 10^5^	3.7139 × 10^5^	3.1010	7.7957	4.0286	0.9469
		patient 2	NS	5.2974			6.7209 × 10^5^	7.8319 × 10^5^	1.9805	6.8742	2.2529	0.9949
			AS		10.0341	2.3349	6.7209 × 10^5^	7.8319 × 10^5^	1.9138	7.8943	2.3814	0.9949
		patient 3	NS	3.6412			1.7133 × 10^5^	1.9739 × 10^5^	2.2544	5.7490	2.4501	0.9708
			AS		7.9745	3.0193	1.7141 × 10^5^	1.9739 × 10^5^	2.1541	6.3284	2.7785	0.9713
		patient 4	NS	4.5396			2.2585 × 10^4^	2.0012 × 10^4^	2.2452	6.2417	2.5590	0.9888
			AS		6.9450	1.9621	2.2589 × 10^4^	2.0012 × 10^4^	2.1415	7.4055	2.5296	0.9888
		patient 5	NS	2.0543			4.6104 × 10^9^	7.6978 × 10^9^	3.1775	6.9649	3.2438	0.8110
			AS		8.7712	8.3197	4.7642 × 10^9^	7.6978 × 10^9^	3.0597	5.8517	4.0535	0.8381
		patient 6	NS	1.9183			2.0410 × 10^6^	2.0939 × 10^6^	3.6680	7.1460	3.7643	0.7730
			AS		5.2350	5.7006	2.1434 × 10^6^	2.0939 × 10^6^	3.4649	6.1130	4.4482	0.8118
	average		NS	3.4818			3.8451 × 10^8^	6.4182 × 10^8^	2.4510	5.8449	2.6399	0.9091
			AS		7.7827	4.4353	3.9734 × 10^8^	6.4182 × 10^8^	2.3359	6.1714	2.9131	0.9230

#### 3.1.1. Basic reproduction ratio, *R*_0_

In the TV model, the basic reproduction ratio is given by 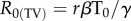. In our study, the within-host *R*_0_ has been estimated to be approximately 3.5 (table 4). *R*_0_ is highly dependent on the model choice and the assumptions about the immune responses following infection. Moderate to high values of *R*_0_ are expected in influenza A as the virus disseminates very rapidly within the host.

#### 3.1.2. Viral growth rate*, r*_0_

Assuming that at the initial stage of the infection the number of target cells remains constant, it can be shown that the initial viral growth rate in the TV model can be described very well by *rβT*_0_ − *γ*. The rate at which the virus population grows in the patient over the initial period of infection is essentially independent of *V*_0_. Typically, the viral growth rate of influenza A is high, suggesting a high value of *R*_0_ [7]. In our study, the average *r*_0_ has been calculated to be approximately equal to 7.8 day^−1^ (table 4).

#### 3.1.3. Generation time, *T*_g_

An approximate solution for the average generation time (at the population level) during the course of the infection for the TIV and TV models is derived in the electronic supplementary material, S3F. Increasing the infection rate, the viral production rate, the initial number of target cells or the initial viral load yields lower *T*_g_ values. When *R*_0_ is relatively large, the increase in the viral clearance rate and the death rate of infected cells also results in lower values for *T*_g_. Moreover, as *R*_0_ decreases and the number of cells that become infected decreases, the increase in these two rates yields higher *T*_g_ values. The TV model predicts an average generation time of influenza A infection at the population level of around 2.6 days (table 4). This agrees with previous estimations using different datasets [9,53].

#### 3.1.4. Area under the viral load curve, *A*_V_

In the electronic supplementary material, S3A, a formula for the area under the viral load curve, *A*_V_, in the target cell-limited models TIV and TV is derived. From the approximation derived, it is clear that the basic reproduction ratio is the main determinant of the value of *A*_V_. In the TV model, as *β* increases *A*_V_ increases exponentially and converges to (*V*_0_ + *rT*_0_)/*γ*, which corresponds to the maximum *A*_V_ (further increase is not possible due to the depletion of susceptible cells). *A*_V_ increases almost linearly with the viral production rate (especially when the parameter values are such that all susceptible cells eventually get infected) and *T*_0_, and it has an inverse relationship with the viral clearance rate and the death rate of infected cells. *A*_V_ is not affected much by varying *V*_0_ as long as *V*_0_ remains low (as will normally be the case in practice).

#### 3.1.5. Peak viral load, *P*_V_

An exact formula of the peak viral load in the TV model, *P*_V(TV)_, is derived in the electronic supplementary material, S3C (an approximation of *P*_V(TIV)_ is also derived in the electronic supplementary material, S3C). From the solution of *P*_V(TV)_, it can be observed that, although the peak viral load is affected by all model parameters, its value is mainly influenced by the viral production rate *r*.

As the infection rate *β* increases, *P*_V_ increases and converges to a constant, which corresponds to the maximum viral load when all the cells are already infected before the start of the decay phase. If *P*_V_ reaches this value, then any antiviral treatment for the protection of susceptible cells that is administered after the time of the peak will have very little or no effect during the viral decay period. By contrast, *P*_V_ increases linearly with the viral production rate and *T*_0_. Hence, an infection by a viral strain with a high replication rate might be more severe than an infection with a higher cell infection rate (see also [12]). Therefore, depending on the other parameter values, therapeutic interventions to reduce viral replication will be more effective than those that reduce viral cell infectivity. Similarly, therapeutic, or prophylactic, interventions that aim at the limitation of target cells, e.g. by inducing resistance to virus, may be more effective. *P*_V_ has an inverse relationship with the viral clearance rate (and the death rate of infected cells in the TIV model, with the increase of the first being slightly more important than that of the second). There is almost no variation of *P*_V_ with *V*_0_.

#### Time to peak viral load, 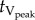


3.1.6.

An approximation of the time to the peak of viral load for the TIV and TV models is given in the electronic supplementary material, S3D. 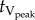 decreases as the infection rate, the viral production rate and the initial number of susceptible cells increase. In cases where the virus disseminates quickly and infects the majority of susceptible cells, a decrease of 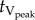 is also observed with increasing viral clearance and infected cell death rates. Otherwise, after a certain value, an increase in these rates delays the onset of the infection and therefore the time to peak. The decrease of *V*_0_ also yields higher 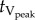. An infection where the peak viral load occurs either much earlier or later is not necessarily a more severe infection and needs to be considered with other measures of infection severity such as the peak viral load and the area under the viral load curve. For example, in table 2 it can be observed that the late occurrence of the peak viral load does not imply a severe infection with respect to the value of the peak. In the standard influenza A virus kinetic pattern, viral load reaches its maximum level approximately 2 days after the initiation of the infection [53], which is in good agreement with our model prediction (table 4).

#### 3.1.7. Viral decay rate*, r*_d_

A derivation of an approximate solution of the viral load during the decay phase for the TIV model is shown in [14] (see also [54]). In the TV model, after the infection of all target cells, the viral load decreases exponentially at rate *γ* (in the late decay phase the main or the only process that takes place is the clearance of the virus). This clearance rate is a composite of the natural death rates of infected cells and free virus and the action of the immune system in enhancing the clearance of both. Although all model parameters can affect the early phase of virus growth, the decay of the virus at the later stage of the infection depends almost exclusively on the death rate of infected cells and the virus clearance rate (in the TIV model, the parameter that has the lower value dominates towards the end of the infection [14,54]). This dependence suggests that a treatment acting on the infection rate and/or the viral production rate will only be effective if it is administered at the early stage of the infection. A treatment administered after the peak viral load will be most effective if it acts on the death rate of infected cells and/or the viral clearance rate. The average viral decay rate in this study was calculated to be approximately 4.4 day^−1^ (table 4).

#### 3.1.8. Duration of infection, *t*_d_

An approximate solution of the duration of infection in the TV model is derived in the electronic supplementary material, S3E. Increasing the infection rate and the number of target cells results in the increase of the basic reproduction ratio and the rapid dissemination of the virus. The rapid infection of the target cells results in the fast convergence to the infection-free steady state where viral load falls to zero. When the infection rate gets too large, a further increase has no significant effect on the infection duration, which then corresponds to the minimum time needed for the infection of all susceptible cells, their death and the clearance of the virus. A similar situation is observed when the initial number of target cells gets very large. An increase in the viral production rate also decreases the infection duration. However, when the rate of virus production is very large, further increase yields higher durations of infection, as then all (or almost all) cells get infected quickly. In this case, the increase in the rate at which free virus is produced by each infected cell results in increased time needed for virus clearance. For relatively large values of *R*_0_, the infection duration also decreases as the viral clearance rate and the death rate of infected cells increase. After a certain value and as *R*_0_ decreases, the virus disseminates at lower rates and, although a significant decrease in peak viral load and the number of dead cells is observed, the time needed for the virus to be cleared increases due to the high availability of susceptible cells. Therefore, based on the simple models considered, a long infection is not necessarily a severe infection. The observed increase in the infection duration with the decrease of the basic reproduction ratio is counter-intuitive, as an infection with low rates of transmissibility between cells should last for less time. This might be considered a limitation of models without an explicit description of immune responses. The TV model predicts that the infection duration is about 5.8 days (table 4), which is approximately the infectious period of a typical influenza A infection recorded in clinical studies where the date of infection is known (household transmission studies [55]).

#### 3.1.9. Fraction of dead cells at the end of the infection, *D*

In the electronic supplementary material, S3B, we derive an approximate solution of *D* for both the TIV and TV models. The solutions of *D* also show that *R*_0_ is the main determinant of the fraction of dead cells at the end of the infection. In particular, decreasing the infection rate, the viral production rate, the initial number of target cells or the initial viral load decreases the total level of cell death due to infection. A decrease in the number of dead cells can also be achieved by increasing the viral clearance rate and the death rate of infected cells. It can be concluded that, although infections with lower infection and virus production rate can be less severe with respect to the peak viral load and the damage they cause to the tissue, they can last longer. The models considered in this paper predict that the majority of epithelial cells will die due to infection (table 4).

### 3.2. Problems in the parameter estimation due to limitations in data availability

Clinical data availability limits the development and validation of more complex models of acute viral infections that can describe the immunological mechanisms and cellular dynamics in more detail. The question therefore arises of whether the quality and quantity of available viral load data are sufficient to support even the simplest models developed to predict viral dynamics and the impact of therapeutic interventions.

#### 3.2.1. Quantity of viral load measurements

For the simplest within-host viral dynamics mathematical model (2.5)–(2.7), at least five viral load measurements at distinct time points (ideally at least one in each day) are required to estimate the unknown model parameters [47]. In this study, 174 out of 191 individual patients were excluded from the analysis because of either the limited number of viral load measurements made in the clinical observation periods post infection or the large number of missing data points (for example, the data points during the initial phase of the infection). The maximum number of viral load measurements in one patient was eight in 7 days of infection, which is sufficient for making predictions but not entirely adequate for deriving precise parameter estimates and estimates of the infection-related quantities. As illustrated in figure 3, more measurements should be taken during the nonlinear phase, around the peak viral load, where in most cases high uncertainty occurs. Two (precise) viral load measurements at distinct time points in each of the initial viral growth and late viral decay phases might be enough to accurately approximate the rates of increase and decrease of the viral load curve. However, although the durations of these phases have been estimated [54], these vary between patients, even within the fairly homogeneous sample of patients with respect to age used in parameter estimation (table 4 and figure 5).

#### 3.2.2. Precision of viral load measurements

In no dataset was there information about the error in the sampling and measurement methods. Consequently, this introduces a degree of uncertainty regardless of the model accuracy to describe given data (and, thus, more frequent measurements alone will not necessarily reduce the uncertainty in the model predictions, figure 3). Ideally, replicating both the viral load assays and sampling at one time point from the same patient would enable us to estimate the error in the measurement of viral load and improve the model predictions. Hence, given that we take the minimum number of measurements required to estimate the parameters (five in the TV model), it might be better to acquire replicate measurements and get more precise values of the viral load instead of taking samples more frequently at different time points. Ideally, both should be done in clinical epidemiological studies of acute infections. However, we acknowledge that, apart from the practical issues in taking multiple samples from the patients in time points close to each other, other sources of variance might affect the accuracy of the measured viral load. For example, the virus shed by patients may differ at different times of the day.

Note that, in the absence of independent information about parameter values, more precise viral load measurements alone will not necessarily reduce the uncertainty of parameter estimates due to the correlations between them (see §3.2.3).

#### 3.2.3. Availability of data other than viral load

Although the model describes the viral load data well, the absence of information about the values of key biological parameters and the dynamics of uninfected and infected cells makes parameter estimation difficult. The high correlation between the model parameters results in equivalent predictions of the model for a wide parameter space. The individual posterior medians and the corresponding 95% credible intervals for the four unknown parameters of the TV model are summarized in the electronic supplementary material, table S5. In the electronic supplementary material, figure S4.2, we provide plots of the posterior distributions of each of these parameters.

The correlations between the parameters for each patient are illustrated by pairwise scatter plots of the parameters (electronic supplementary material, figures S4.3–S4.8). There is correlation between almost all parameters. In particular, in most of the cases the virus clearance rate *γ* is inversely correlated with parameter *β* and changes linearly with parameter *l*, while *l* is inversely proportional to *β*. Although the relationships of *β*, *γ* and *l* with the initial viral load *V*_0_ are not clear, it seems that *V*_0_ is inversely proportional to *β* and *l* and not particularly related to *γ*. Moreover, variation in *V*_0_ does not influence the model fit much, unless *V*_0_ varies from very small to very large values. The correlations between the model parameters are not surprising as virus infects cells and in turn infected cells produce more virus. Therefore, the virus dynamics can be controlled by just protecting the cells not yet infected, which can be achieved either by decreasing the infection rate or the rate at which virus is produced by each infected cell or by increasing either the killing rate of infected cells or the clearance rate of the virus. Independent measurements of some unknown model parameters would therefore help to produce more precise estimates of the remaining parameters.

Despite the high uncertainty in the estimates of the values of single parameters, their product may be estimated more precisely, resulting in the derivation of more accurate estimates of some of the infection-related quantities. For example, figure S4.1 in the electronic supplementary material S4 illustrates that the basic reproduction ratio *R*_0_ can be estimated precisely irrespective of the high uncertainty in individual parameter estimates (*R*_0_ = *lT*_0_/*γ* is eventually equal to the slope of the line showing the linear relationship between *l* and *γ*, illustrated in the electronic supplementary material, figure S4.7, multiplied by the initial number of target cells *T*_0_).

#### 3.2.4. Unknown lower limit of quantification

The different viral load assays differ in the reported lower limits of quantitation (LLOQ) and detection. In our analysis, we had no information on the LLOQ and set it to 0.7 TCID_50_ ml^−1^. However, the choice of this value might significantly influence both the model fit and the uncertainty in the parameter estimates.

#### 3.2.5. Unknown sampling time

During our study, the exact sampling time during a day in the data we used was unknown. Given the available information, we assumed that in the Roche dataset samples were taken every 0.5 days between days 1 and 3 and every 1 day afterwards. In the GSK dataset, we assumed that samples were taken exactly every 24 h. However, the precise sampling times can significantly affect the model predictions and therefore they should be recorded. Recording whether the sample was taken in the morning or afternoon is not enough, as variations in the sampling time by some hours might be influential.

## 4. Discussion

In the past few decades, mathematical model development and its use in the study of infectious diseases in general, and influenza A in particular, has largely focused on disease spread and control within populations of hosts [56]. Models of viral dynamics within an individual person serve many purposes, including creating a better understanding of what determines the temporal trajectory of viral load over time, identifying what needs to be measured experimentally and facilitating the choice of endpoints in clinical trials of possible therapies. Precise mathematical description of viral dynamics also facilitates understanding of the immune response and the assessment of the efficacy of antiviral treatments. It also plays an indirect role in understanding virus transmission between hosts, due to the relationship between viral load and infectiousness, and it can help in the evaluation of therapeutic treatments as part of the development of effective strategies for the mitigation and control of epidemics.

Mathematical models with varying complexity have been developed and analysed by analytical and numerical approaches to provide a description of the growth and decay of the influenza A virus within the patient. However, gaps in knowledge, especially concerning the role of different immune system components and the lack of detailed biological data on, for example, the life expectancies of cells and free virus at different stages of infection, limit model validation.

In this paper, we revisited and extended simple classic models of viral kinetics to study the influenza A infection within the human body. Despite their simplicity, models that exclude the explicit representation of the immune responses can adequately explain observed patterns of viral growth and decay in patients and facilitate an understanding of the processes that have the greatest impact on the course of infection. One of the advantages of using simple models to describe the infection dynamics is that a number of quantities that reflect the severity of the infection, and some of them can be considered as clinical endpoints used in the assessment of treatments, can be derived analytically. Owing to the variability of the measurements of viral load, and clinical outcome, as well as the limited number of measurements, the derivation of reliable estimates of such quantities directly from the raw data would be difficult. We derived approximate expressions and estimations of a series of morbidity and viral growth and decay related measures, such as the area under the viral load curve, the duration of infection, time to peak viral load and the slope of the viral decay curve, and focused on identifying their key determinants. New results are presented on some of these measures. This focus is a first step in developing tools to aid in the design of clinical trials of candidate therapies to treat infected patients or susceptible individuals to lessen the impact of infection. Despite the limited information that each of these quantities can provide independently, and difficulties in measuring them, the assessment of all quantities together provides insights into how different interventions will act on the observed course of infection when applied at different times post infection and post the initiation of treatment. Most importantly, they also help define what to measure in clinical trials of therapies.

Although we focused on influenza A virus infection, the models and results are applicable to other acute viral infections where measurable viral load persists for a few days to a few weeks in the patient. The fast dynamics of such infections and the short duration of viral replication create problems in the design and conducting of quantitative clinical studies of possible therapies involving sampling from infected patients. However, problems in the estimation of the model parameters and the accurate prediction of the viral load dynamics can be resolved by improving data quality. Based on our analysis, we suggest that frequent sampling at defined time points is essential to creating a deeper understanding of viral kinetics and between-patient variation. More frequent viral load measurements will be more useful if they are accompanied by measurement error data. Reduction of the measurement error of viral load could be achieved by running replicate measurements and replicating sampling of the same patient at one time point. This reduction in the measurement error will also be very important in using these models to guide the design of clinical trials of therapeutic interventions. Determining the importance of variance in viral load between patients and during therapy requires understanding the variation inherent in the sampling and measurements methods. Describing these two sources of variation (between patients and within the sampling and measurement method) is essential in the determination of the efficacy of a therapeutic intervention.

Improving the precision of viral load measurements alone will not necessarily result in more accurate parameter estimates. A major need in future clinical, animal model and *in vitro* studies is the determination of basic population dynamic parameters, such as cell life expectancies with and without infection and the virus clearance rate. At present their accurate estimation from the time course of infection in the patient is fraught with difficulty, due to the correlations between them, and independent measurements of at least some of them is required.

## Supplementary Material

Supplementary material S1

## Supplementary Material

Supplementary material S2

## Supplementary Material

Supplementary material S3

## Supplementary Material

Supplementary material S4

## Supplementary Material

Supplementary material S5

## Data accessibility

Raw data are available from https://clinicalstudydatarequest.com for researchers who meet the criteria for access to confidential data.
