# Supplementary material for: Understanding the within-host dynamics of influenza A virus: from theory to clinical implications
Source: J R Soc Interface. 2016 Jun;13(119):20160289. doi: 10.1098/rsif.2016.0289 (PMC4938090; doi:10.1098/rsif.2016.0289)
Supplement: Supplementary material S1 [file rsif20160289supp1.pdf]

Supplementary material S1. The TVA model

S1A The TVA model

We extend the TV model to include a representation of the overall action strength of the immune response against influenza A. The immune response, represented by the variable A, is assumed to be activated and increased proportionally to the viral load with rate α. It then clears free virus particles and reduces the viral load at rate wAV, where w is the instantaneous per virus loss coefficient as a function of A. The decay of A during the infection is considered negligible. Moreover, including a constant decay rate into the model does not improve much the model fit (data not provided). In addition, time delays in the activation and the action of the immune response are also not considered (but in some cases these might be important [1, 2]). The dynamics of this system are governed by the following differential equations:

$$\frac{dT}{dt} = -\beta VT, \quad \text{(S1A.1)}$$

$$\frac{dV}{dt} = r\beta VT - \gamma V - wAV, \quad \text{(S1A.2)}$$

$$\frac{dA}{dt} = aV, \quad \text{(S1A.3)}$$

with initial conditions

$$T(0) = T_0, V(0) = V_0, A(0) = A_0. \quad \text{(S1A.4)}$$

S1B Rescaling the TVA model

Let $T = a_1\tilde{T}, V = a_2\tilde{V}, A = a_3\tilde{A}, t = a_4\tau$.

$$\frac{a_1 d\tilde{T}}{a_4 d\tau} = -\beta a_1 a_2 \tilde{T}\tilde{V}, \quad \text{(S1B.1)}$$

$$\frac{a_2 d\tilde{V}}{a_4 d\tau} = r\beta a_1 a_2 \tilde{T}\tilde{V} - a_2\gamma\tilde{V} - wa_2 a_3 \tilde{V}\tilde{A}, \quad \text{(S1B.2)}$$

$$\frac{a_3 d\tilde{A}}{a_4 d\tau} = aa_2\tilde{V}. \quad \text{(S1B.3)}$$

Let $a_1 = \frac{\tilde{r}}{r}, a_2 = 1, a_3 = \frac{a}{\tilde{a}}, a_4 = 1$, where $\tilde{r}$ and $\tilde{a}$ are arbitrary values. Then, the TVA model reduces to the following one:

$$\frac{d\tilde{T}}{dt} = -\beta\tilde{T}\tilde{V}, \qquad \text{(S1B.4)}$$

$$\frac{dV}{dt} = \tilde{r}\beta\tilde{T}\tilde{V} - \gamma\tilde{V} - w\frac{a}{\tilde{a}}\tilde{V}\tilde{A}, \qquad \text{(S1B.5)}$$

$$\frac{d\tilde{A}}{dt} = \tilde{a}\tilde{V}. \qquad \text{(S1B.6)}$$

Hence, if (T^*, V^*, A^*) is a solution of the TVA model with parameters (β, r, γ, w, a) and initial conditions (T_0, V_0, A_0), then $(\tilde{T}^*, \tilde{V}^*, \tilde{A}^*) = \tilde{r}T^*/r, V^*, aA^*/\tilde{a}$ is the solution of the TVA model with parameters $(\beta, \tilde{r}, \gamma, wa/\tilde{a}, \tilde{a})$ and initial conditions $(\tilde{r}T_0/r, V_0, aA_0/\tilde{a})$.

References

1. Baccam P, Beauchemin C, Macken CA, Hayden FG, Perelson AS. Kinetics of influenza A virus infection in humans. Journal of virology. 2006;80(15):7590-9.
2. Handel A, Longini IM, Jr., Antia R. Towards a quantitative understanding of the within-host dynamics of influenza A infections. Journal of the Royal Society, Interface / the Royal Society. 2010;7(42):35-47.
