# Supplementary material for: Understanding the within-host dynamics of influenza A virus: from theory to clinical implications
Source: J R Soc Interface. 2016 Jun;13(119):20160289. doi: 10.1098/rsif.2016.0289 (PMC4938090; doi:10.1098/rsif.2016.0289)
Supplement: Supplementary material S2 [file rsif20160289supp2.pdf]

Supplementary material S2. Infection related quantities

Basic reproduction ratio, R_0, viral growth rate, r_0, and generation time, T_g. The basic reproduction ratio, R_0, characterises the early stage of infection. In the context of viral dynamics this is defined as the average number of newly infected cells that arise from an infected cell during its infectious period when introduced into a population of entirely susceptible cells. This is a crucial quantity which determines whether virus will grow and establish an infection, which happens when $R_0 > 1$. Note that R_0 is a composite parameter with all the reproduction and infection processes in the numerator and all the loss terms in the denominator. As such its important role in the dynamics of viral growth and decay underlies the close associations between parameters and hence the concomitant difficulty in their estimation from measures only of changes in viral load over time post infection in individual patients.

The initial viral growth rate, r_0, characterises the speed of initial viral load expansion.

Another interesting quantity is the generation time within an individual host, which is defined as the mean time between the infection of a cell and the infection of secondary cases by that cell. The basic reproduction ratio, the initial viral growth rate and the generation time within host dictate the speed at which the virus disseminates within the human host (and concomitantly, symptom appearance plus associated morbidity) and therefore the speed at which therapeutic interventions should be applied to reduce the amount of viral shedding. The three quantities are related [1]. In this paper we are particularly interested in deriving and estimating the generation time at the population level T_g from viral load data. T_g is defined as the mean time interval between an individual becoming infected and the infection of the other persons that individual infects [2].

Area under the viral load curve, A_V. This is the total amount of virus shed during the infection course and reflects the infectiousness of the host. In particular, a person is thought to be infectious when the viral load is above a given threshold, and the area under the viral load curve is defined as the area between the curve of the viral load and that threshold [3, 4]. Reducing A_V with treatment will not only help the patient but will also reduce the probability of the disease spreading between people. This quantity has been frequently used as the primary endpoint measure in clinical trials of candidate anti-viral therapies.

Peak viral load, P_V. This is thought to be correlated with symptoms scores such as body temperature.

Time to peak viral load, t_{Vpeak}. This quantity can be used for assessing infection progression and designing interventions with antiviral drugs. When antiviral drugs (at least those currently available, e.g. neuraminidase inhibitors) are administered after the peak viral load, they are likely to have a limited effect. Thus this time point can be used as an estimation of the time limit by which the use of therapeutic interventions would be most effective (see [5, 6]). In [7] it has been shown that the highest total symptoms score occurs approximately one day after the peak of viral shedding.

Viral decay rate, r_d. This is the rate of viral clearance and it is an important indicator of the impact of any treatment given after the peak viral load has occurred.

Duration of infection, t_d. This is defined as the time interval in which the viral load is above a critical value, which is the value below which the virus cannot be detected (virus detection limit). This quantity helps to distinguish short-lived infections from more severe or chronic infections where other factors (e.g. immunodeficiency) may determine the course of viraemia [4]. It can also be used

as a measure of the time a patient is infectious, and the time required in isolation or quarantine to prevent onward infection.

Fraction of dead cells at the end of the infection, D. This can be used to assess the likelihood of clinical symptoms and associated morbidity. This quantity indicates the damage to the tissue caused by the infection, and therefore the complications that patients may experience, the occurrence of some respiratory symptoms that result from direct cytopathic effects of the virus [6, 8], and the time required for the recovery of the patient [4]. D is difficult to measure and experimental data to support the estimation of this quantity is limited [9-12].

References

1. Wallinga J, Lipsitch M. How generation intervals shape the relationship between growth rates and reproductive numbers. Proceedings of the Royal Society B: Biological Sciences. 2007;274(1609):599-604.
2. Svensson Å. A note on generation times in epidemic models. Mathematical Biosciences. 2007;208(1):300-11.
3. Canini L, Carrat F. Population modeling of influenza A/H1N1 virus kinetics and symptom dynamics. Journal of virology. 2011;85(6):2764-70.
4. Dobrovolny HM, Reddy MB, Kamal MA, Rayner CR, Beauchemin CA. Assessing mathematical models of influenza infections using features of the immune response. PloS one. 2013;8(2):e57088.
5. Lee HY, Topham DJ, Park SY, Hollenbaugh J, Treanor J, Mosmann TR, et al. Simulation and prediction of the adaptive immune response to influenza A virus infection. Journal of virology. 2009;83(14):7151-65.
6. Dobrovolny HM, Baron MJ, Gieschke R, Davies BE, Jumbe NL, Beauchemin CA. Exploring cell tropism as a possible contributor to influenza infection severity. PloS one. 2010;5(11):e13811.
7. Carrat F, Vergu E, Ferguson NM, Lemaitre M, Cauchemez S, Leach S, et al. Time lines of infection and disease in human influenza: a review of volunteer challenge studies. American journal of epidemiology. 2008;167(7):775-85.
8. Hayden FG, Fritz R, Lobo MC, Alvord W, Strober W, Straus SE. Local and systemic cytokine responses during experimental human influenza A virus infection. Relation to symptom formation and host defense. The Journal of clinical investigation. 1998;101(3):643-9.
9. Saenz RA, Quinlivan M, Elton D, Macrae S, Blunden AS, Mumford JA, et al. Dynamics of influenza virus infection and pathology. Journal of virology. 2010;84(8):3974-83.
10. Schulze-Horsel J, Schulze M, Agalaridis G, Genzel Y, Reichl U. Infection dynamics and virus-induced apoptosis in cell culture-based influenza vaccine production—Flow cytometry and mathematical modeling. Vaccine. 2009;27(20):2712-22.
11. Iwasaki T, Nozima T. Defense mechanisms against primary influenza virus infection in mice. I. The roles of interferon and neutralizing antibodies and thymus dependence of interferon and antibody production. Journal of immunology (Baltimore, Md : 1950). 1977;118(1):256-63.
12. Handel A, Longini IM, Jr., Antia R. Towards a quantitative understanding of the within-host dynamics of influenza A infections. Journal of the Royal Society, Interface / the Royal Society. 2010;7(42):35-47.
