# Supplementary material for: Understanding the within-host dynamics of influenza A virus: from theory to clinical implications
Source: J R Soc Interface. 2016 Jun;13(119):20160289. doi: 10.1098/rsif.2016.0289 (PMC4938090; doi:10.1098/rsif.2016.0289)
Supplement: Supplementary material S3 [file rsif20160289supp3.pdf]

Supplementary material S3. Derivation of the results

We present the results we derived for the TIV and TV models.

S3A Area under the viral load curve

The area under the viral load curve during the course of the infection, A_V, is given by

$$A_V = \int_{t_{d_1}}^{t_{d_2}} V dt, \quad \text{(S3A.1)}$$

where t_{d_1} and t_{d_2} are the times at which the viral load takes the value of the detection limit at the beginning and the end of the infection (or $t_{d_2} - t_{d_1}$ can be defined as the duration in which the patient is infectious). If the initial viral load is above the detection limit then t_{d_1} is considered as the time at which virus enters the body.

TIV model

From (1) and (2) we get,

$$I = -\frac{1}{\delta_I}\frac{dT}{dt} - \frac{1}{\delta_I}\frac{dI}{dt}. \quad \text{(S3A.2)}$$

Substituting (S3A.2) into (3) and integrating from $t = t_{d_1}$ to $t = t_{d_2}$ we get

$$\int_{t_{d_1}}^{t_{d_2}} \frac{dV}{dt} dt = -\frac{p}{\delta_I}\int_{t_{d_1}}^{t_{d_2}} \frac{dT}{dt} dt - \frac{p}{\delta_I}\int_{t_{d_1}}^{t_{d_2}} \frac{dI}{dt} dt - c\int_{t_{d_1}}^{t_{d_2}} V dt \quad \text{(S3A.3)}$$

$$\Rightarrow A_{V(TIV)} = \frac{1}{c}\left[-\frac{p}{\delta_I}\left(T_{t_{d_2}} - T_{t_{d1}} + I_{t_{d_2}} - I_{t_{d1}}\right) + V_{t_{d1}} - V_{t_{d_2}}\right], \quad \text{(S3A.4)}$$

where $T_{t_{d_1}} = T(t_{d_1}), T_{t_{d_2}} = T(t_{d_2}), I_{t_{d_1}} = I(t_{d_1}), I_{t_{d_2}} = I(t_{d_2}), V_{t_{d_1}} = V(t_{d_1}), V_{t_{d_2}} = V(t_{d_2})$.

Integrating (1) from $t = t_{d_1}$ to $t = t_{d_2}$ we get

$$T\left(t_{d_2}\right) = T\left(t_{d_1}\right)e^{-\beta A_{V(TIV)}}. \quad \text{(S3A.5)}$$

Substituting (S3A.5) into (S3A.4) we get

$$A_{V(TIV)} = \frac{1}{c}\left[-\frac{p}{\delta_I}\left(T\left(t_{d_1}\right)e^{-\beta A_{V(TIV)}} - T_{t_{d1}} + I_{t_{d_2}} - I_{t_{d1}}\right) + V_{t_{d1}} - V_{t_{d_2}}\right]. \quad \text{(S3A.6)}$$

Hence, the area under the viral load curve in the TIV model, $A_{V(TIV)}$, is given by the solution of equation (S3A.6). An expression for $A_{V(TIV)}$ can be obtained by using the Lambert function [1, 2], which cannot by expressed in terms of elementary functions.

Multiplying (S3A.6) by β and rearranging we get

$$\beta A_{V(TIV)} - \frac{\beta p}{c\delta_I} T_{t_{d1}} + \frac{\beta p}{c\delta_I} I_{t_{d_2}} - \frac{\beta p}{c\delta_I} I_{t_{d1}} + \frac{\beta}{c} V_{t_{d_2}} - \frac{\beta}{c} V_{t_{d1}} = -\frac{\beta p}{c\delta_I} T(t_{d_1}) e^{-\beta A_{V(TIV)}} . \quad \text{(S3A.7)}$$

Let

$$x_1 = \beta A_{V(TIV)} - \frac{\beta p}{c\delta_I} T_{t_{d1}} + \frac{\beta p}{c\delta_I} I_{t_{d_2}} - \frac{\beta p}{c\delta_I} I_{t_{d1}} + \frac{\beta}{c} V_{t_{d_2}} - \frac{\beta}{c} V_{t_{d1}} . \quad \text{(S3A.8)}$$

From (S3A.7) and (S3A.8) we obtain

$$x_1 e^{x_1} = -\frac{\beta p}{c\delta_I} T(t_{d_1}) e^{-\frac{\beta p}{c\delta_I} T_{t_{d_1}} + \frac{\beta p}{c\delta_I} I_{t_{d2}} - \frac{\beta p}{c\delta_I} I_{t_{d_1}} + \frac{\beta}{c} V_{t_{d2}} - \frac{\beta}{c} V_{t_{d_1}}} . \quad \text{(S3A.9)}$$

Let

$$z_1 = -\frac{\beta p}{c\delta_I} T(t_{d_1}) e^{-\frac{\beta p}{c\delta_I} T_{t_{d_1}} + \frac{\beta p}{c\delta_I} I_{t_{d2}} - \frac{\beta p}{c\delta_I} I_{t_{d_1}} + \frac{\beta}{c} V_{t_{d2}} - \frac{\beta}{c} V_{t_{d_1}}} . \quad \text{(S3A.10)}$$

$z_1 \in \mathbb{R}$ and $-\frac{1}{e} < z_1 \leq 0$.

$x_1 = w(z_1)$ is the upper branch of the Lambert W function, i.e. $w(z_1)e^{w(z_1)} = z_1$.

Hence, from (S3A.8)

$$A_{V(TIV)} = \frac{1}{\beta}\left(w(z_1) + \frac{\beta p}{c\delta_I} T_{t_{d_1}} + \frac{\beta p}{c\delta_I} I_{t_{d_1}} - \frac{\beta p}{c\delta_I} I_{t_{d_2}} + \frac{\beta}{c} V_{t_{d_1}} - \frac{\beta}{c} V_{t_{d_2}} \right) . \quad \text{(S3A.11)}$$

To approximate (S3A.11) we can assume that $T_{t_{d_1}} \approx T_0$, $I_{t_{d_1}} \approx I_0$, $I_{t_{d_2}} \approx 0$, $V_{t_{d_1}} = V_{t_{d_2}}$ and $T_0 >> I_0$:

$$A_{V(TIV)\text{App1}} = \frac{1}{\beta}\left(w(z_1) + \frac{\beta p}{c\delta_I} T_0 \right), \text{ with } z_1 \approx -\frac{\beta p}{c\delta_I} T_0 e^{-\frac{\beta p}{c\delta_I} T_0} . \quad \text{(S3A.12)}$$

In terms of the basic reproduction ratio, $R_0 = \beta p T_0 / c\delta_I$, $A_{V(TIV)\text{App1}}$ is given by

$$A_{V(TIV)\text{App1}} = \frac{1}{\beta}\left(w(z_1) + R_0 \right), \quad \text{with } z_1 \approx -R_0 e^{-R_0} . \quad \text{(S3A.13)}$$

The Taylor series of the upper branch of the W function is given by

$$w(z_1) \quad = \quad z_1 - z_1^{\ 2} + \frac{3}{2} z_1^{\ 3} - \frac{8}{3} z_1^{\ 4} + \dots \tag{S3A.14}$$

Since $-\frac{1}{e} < z_1 \leq 0$, although $1/\beta$ is typically a very big number, the area under the viral load curve can be approximated very well by taking up to the second order terms of the Taylor expansion of $w(z_1)$. This results in the following approximation of $A_{V(TIV)}$:

$$A_{V(TIV)\text{App2}} \quad = \quad \frac{R_0}{\beta}\left(1 - e^{-R_0} - R_0 e^{-2R_0}\right). \tag{S3A.15}$$

Note that in the case where all the target cells die due to the infection, that is $T_{t_{d_2}} = 0$, then from (S3A.4) we obtain

$$A_{V(TIV),T_{t_{d_2}}=0} \quad = \quad \frac{1}{c}\left[V_0 + \frac{p}{\delta_I}\left(T_0 + I_0\right)\right]. \tag{S3A.16}$$

TV model

As in [2], from equation (5)

$$\frac{dT}{dt} \quad = \quad -\beta VT \Rightarrow VT = -\frac{1}{\beta}\frac{dT}{dt}. \tag{S3A.17}$$

Substituting (S3A.17) into (6) and integrating from $t = t_{d_1}$ to $t = t_{d_2}$ we get

$$\int_{t_{d_1}}^{t_{d_2}} \frac{dV}{dt} dt \quad = \quad -r \int_{t_{d_1}}^{t_{d_2}} \frac{dT}{dt} dt - \gamma \int_{t_{d_1}}^{t_{d_2}} V dt \tag{S3A.18}$$

$$\Rightarrow A_{V(TV)} \quad = \quad \frac{1}{\gamma}\left[-r\left(T(t_{d_2}) - T(t_{d_1})\right) + V(t_{d_1}) - V(t_{d_2})\right]. \tag{S3A.19}$$

As before, from (5) we get

$$T(t_{d_2}) \quad = \quad T(t_{d_1}) e^{-\beta A_{V(TV)}}. \tag{S3A.20}$$

It should be noted that due to the very similar structure of the TV model to that of the SIR model in infectious disease epidemiology, the area under the viral load curve is 'equivalent' to the final epidemic size in the SIR model, which can be obtained by solving a similar expression as (S3A.20) [3, 4].

Substituting (S3A.20) into (S3A.19) we get

$$A_{V(TV)} \quad = \quad \frac{1}{\gamma}\left[-rT(t_{d_1})\left(e^{-\beta A_{V(TV)}} - 1\right) + V(t_{d_1}) - V(t_{d_2})\right]. \tag{S3A.21}$$

Hence, the area under the viral load curve in the TV model, $A_{V(TV)}$, is given by the solution of equation (S3A.21). As in the case of the TIV model, since this cannot be solved using elementary functions, to get an expression of $A_{V(TV)}$ we use the Lambert function [1, 2].
Multiplying equation (S3A.21) by β and rearranging we get

$$\beta A_{V(TV)} - \frac{r\beta}{\gamma}T(t_{d_1}) + \frac{\beta}{\gamma}V(t_{d_2}) - \frac{\beta}{\gamma}V(t_{d_1}) \quad = \quad -\frac{r\beta}{\gamma}T(t_{d_1})e^{-\beta A_{V(TV)}} . \qquad \text{(S3A 22)}$$

Let

$$x_2 \quad = \quad \beta A_{V(TV)} - \frac{r\beta}{\gamma}T(t_{d_1}) + \frac{\beta}{\gamma}V(t_{d_2}) - \frac{\beta}{\gamma}V(t_{d_1}) . \qquad \text{(S3A.23)}$$

Then, from (S3A 22) and (S3A.23) we obtain

$$x_2 e^{x_2} \quad = \quad -\frac{r\beta}{\gamma}T(t_{d_1})e^{-\frac{r\beta}{\gamma}T(t_{d_1}) + \frac{\beta}{\gamma}V(t_{d_2}) - \frac{\beta}{\gamma}V(t_{d_1})} . \qquad \text{(S3A.24)}$$

Let

$$z_2 \quad = \quad -\frac{r\beta}{\gamma}T(t_{d_1})e^{-\frac{r\beta}{\gamma}T(t_{d_1}) + \frac{\beta}{\gamma}V(t_{d_2}) - \frac{\beta}{\gamma}V(t_{d_1})} . \qquad \text{(S3A.25)}$$

$z_2 \in \mathbb{R}$ and $-\frac{1}{e} < z_2 \leq 0$.

$x_2 = w(z_2)$ is the Lambert W function, i.e. $w(z_2)e^{w(z_2)} = z_2$.

Hence, from (S3A.23),

$$A_{V(TV)} \quad = \quad \frac{1}{\beta}\left(w(z_2) + \frac{r\beta}{\gamma}T(t_{d_1}) + \frac{\beta}{\gamma}V(t_{d_1}) - \frac{\beta}{\gamma}V(t_{d_2}) \right) . \qquad \text{(S3A.26)}$$

As in the case of the TIV model, to approximate $A_{V(TV)}$ in (S3A.26) we can assume that $T_{t_{d_1}} \approx T_0$ and $V_{t_{d_1}} = V_{t_{d_2}}$ to obtain:

$$A_{V(TV)\text{App1}} \quad = \quad \frac{1}{\beta}\left(w(z_2) + \frac{r\beta}{\gamma}T_0 \right), \text{ with } z_2 \approx -\frac{r\beta}{\gamma}T_0 e^{-\frac{r\beta}{\gamma}T_0} . \qquad \text{(S3A.27)}$$

In terms of the basic reproduction ratio, $R_0 = \frac{r\beta}{\gamma}T_0$,

$$A_{V(TV)\text{App1}} \quad = \quad \frac{1}{\beta}\left(w(z_2) + R_0 \right), \text{ where } z_2 = -R_0 e^{-R_0} , \qquad \text{(S3A.28)}$$

which is the same as (S3A.13).

S3B Fraction of dead cells at the end of the infection

The fraction of cells that die during the duration in which the virus is above the detection limit, D, is

$$D \quad = \quad 1-\frac{T_{t_{d_2}}}{T_0}, \tag{S3B.1}$$

where $T_{t_{d_2}}$ is the number of susceptible cells at time t_{d_2}.

TIV model

Solving (S3A.4) for $T_{t_{d_2}}$ and using (S3A.11) we obtain that the total number of susceptible cells at time t_{d_2} in the TIV model, $T_{t_{d_2}(TIV)}$, is given by

$$T_{t_{d_2}(TIV)} \quad = \quad -\frac{c\delta_I}{\beta p}w(z_1), \tag{S3B.2}$$

where w is the Lambert function with z_1 given by (S3A.10).

Hence, the fraction of cells that die during the infection in the TIV model, D_{TIV} , is

$$D_{TIV} \quad = \quad 1+\frac{c\delta_I}{\beta p T_0}w(z_1) \;=\; 1+\frac{1}{R_0}w(z_1). \tag{S3B.3}$$

Assuming that at t_{d_1}, $T_{t_{d_1}} = T_0$, $I_{t_{d_1}} \approx I_0$ and $V_{t_{d_1}} \approx V_0$, that t_{d_2} is the time at which all infected cells are killed and the virus is cleared, and so $I_{t_{d_2}} = 0$ and $V_{t_{d_2}} = 0$, and using the second order Taylor's expansion of the W function given by (S3A.14), then the fraction of dead cells at the end of the infection can be approximated by

$$D_{\text{(TIV)App1}} \quad = \quad 1-e^{-\frac{\beta}{c}V_0-R_0\left(1+\frac{I_0}{T_0}\right)}-R_0e^{-2\frac{\beta}{c}V_0-2R_0\left(1+\frac{I_0}{T_0}\right)}. \tag{S3B.4}$$

Given that $\frac{I_0}{T_0}+\frac{\delta_I V_0}{pT_0} << 1$ then D_{TIV} can be approximated by

$$D_{\text{(TIV)App2}} \quad = \quad 1-e^{-R_0}-R_0e^{-2R_0} \quad = \quad \frac{\beta A_{V(TIV)\text{App2}}}{R_0}. \tag{S3B.5}$$

TV model

As before, rearranging equation (S3A.19) and using (S3A.26) we obtain that the number of susceptible cells at the point $t = t_{d_2}$ at which virus reaches the detection limit in the decay phase in the TV model is given by

$$T_{t_{d_2}(TV)} = -\frac{\gamma}{r\beta} w(z_2), \quad \text{(S3B.6)}$$

where w is the Lambert function with z_2 given by (S3A.25). Thus, the fraction of cells that die during the infection in the TV model, D_{TV}, is

$$D_{TV} = 1+\frac{\gamma}{r\beta T_0} w(z_2) = 1+\frac{1}{R_0} w(z_2), \quad \text{(S3B.7)}$$

which is of the same form as (S3B.3). Assuming that $T_{t_{d1}} = T_0$, $V_{t_{d1}} = V_0$, $V_{t_{d_2}} = 0$ and approaching $w(z_2)$ by the second order Taylor's expansion of the W function given by (S3A.14), and given that $\frac{V_0}{rT_0} << 1$, then the fraction of dead cells at the end of the infection can also be approximated by (S3B.5).

S3C Peak viral load

Let the maximum of the viral load be achieved at $t = t_{Vpeak}$.

TIV model

At $t = t_{Vpeak}$, $\frac{dV}{dt} = 0$. From (3) we then get

$$I\left(t_{Vpeak}\right) = \frac{c}{p} V\left(t_{Vpeak}\right). \quad \text{(S3C.1)}$$

Substituting (S3A.2) into (3), integrating from $t = 0$ to $t = t_{Vpeak}$ and using (S3C.1) and (4) we obtain

$$V\left(t_{Vpeak}\right)_{(TIV)} = \frac{\delta_I}{c+\delta_I}\left[V_0 - \frac{p}{\delta_I}\left(T(t_{Vpeak}) - T_0\right) + \frac{c}{\beta}\left(\ln T(t_{Vpeak}) - \ln T_0\right) + \frac{p}{\delta_I} I_0\right]. \quad \text{(S3C.2)}$$

Assume that $T(t_{Vpeak}) = aT_0$. Substituting into (S3C.2) we obtain

$$V\left(t_{Vpeak}\right)_{(TIV)} = \frac{\delta_I}{c+\delta_I}\left[V_0 + \frac{p}{\delta_I} T_0\left(1-a\right) + \frac{c}{\beta}\ln a + \frac{p}{\delta_I} I_0\right]. \quad \text{(S3C.3)}$$

In the examples we have considered in the main text, the average value of a is approximately equal to 0.13.

TV model

In the TV model, from equation (6) at $t = t_{Vpeak}$

$$T\left(t_{Vpeak}\right) \quad = \quad \frac{\gamma}{r\beta}. \qquad \text{(S3C.4)}$$

From equation (5) we get

$$\int_{T_0}^{T(t_{Vpeak})} \frac{dT}{T} \quad = \quad -\int_0^{t_{Vpeak}} \beta V(t)dt \Rightarrow \int_0^{t_{Vpeak}} V(t)dt = -\frac{1}{\beta}\left(\ln T(t_{Vpeak}) - \ln T_0\right) \qquad \text{(S3C.5)}$$

$$\Rightarrow \int_0^{t_{Vpeak}} V(t)dt \quad = \quad \frac{1}{\beta}\ln\frac{r\beta T_0}{\gamma}. \qquad \text{(S3C.6)}$$

Integrating equation (6) from $t = 0$ to $t = t_{Vpeak}$ and using (5) and (S3C.6) we get

$$\int_{V_0}^{V(t_{Vpeak})} dV \quad = \quad -r\int_{T_0}^{T(t_{Vpeak})} dT - \frac{\gamma}{\beta}\ln\frac{r\beta T_0}{\gamma} \qquad \text{(S3C.7)}$$

$$\Rightarrow V\left(t_{Vpeak}\right)_{(TV)} \quad = \quad V_0 + rT_0 - \frac{\gamma}{\beta}\left(\ln\frac{r\beta T_0}{\gamma} + 1\right) = V_0 + rT_0 - \frac{\gamma}{\beta}\left(\ln R_0 + 1\right). \qquad \text{(S3C.8)}$$

S3D Time to peak viral load

TIV model

As shown in previous work [5, 6], assuming that at the initial phase of the infection the number of target cells remains constant, i.e. $T(t) = T_0$, then the initial viral growth rate r_0 can be approximated by the dominant root of the characteristic equation of the Jacobian matrix of the resulting linear system. Hence, the viral load at the initial phase of the infection, $V_{init(t)}$, can be approximated by

$$V_{init}(t) \quad = \quad A\vec{u}e^{r_0 t}, \qquad \text{(S3D.1)}$$

where u is the eigenvector corresponding to r_0 and A is a constant (see [5, 6]). Clearly this is a good approximation as long as $T \approx T_0$. If we assume that the viral load continues to increase linearly until the peak, then using (S3D.1), we can approximate peak viral load by

$$V\left(t_{Vpeak}\right) \quad = \quad c\vec{u}e^{r_0 t_{Vpeak}}. \qquad \text{(S3D.2)}$$

Solving (S3D.2) for t_{Vpeak} we derive an approximation of the time to peak viral load in the TIV model:

$$t_{Vpeak(TIV)} \quad = \quad \frac{\ln V(tpeak) - \ln c\vec{u}}{r_0}. \qquad \text{(S3D.3)}$$

Clearly this will always be an underestimation as the viral growth decelerates when approaching peak viral load.

TV model

Integrating equation (6) from $t = 0$ to $t = t_{Vpeak}$ and rearranging we get

$$t_{Vpeak} = \frac{1}{\gamma}\left(r\beta\int_0^{t_{Vpeak}} T dt + \ln\frac{V_0}{V\left(t_{Vpeak}\right)}\right), \quad \text{(S3D.4)}$$

where $V\left(t_{Vpeak}\right)$ is the maximum viral load in the TV model given by (S3C.8).

Dividing equation (6) by equation (5) we obtain

$$\frac{dV}{dT} = -r + \frac{\gamma}{\beta}\frac{1}{T}. \quad \text{(S3D.5)}$$

Integrating (S3D.5) and using (7) we obtain

$$V(t)_{(TV)} = -rT(t) + \frac{\gamma}{\beta}\ln T(t) + V_0 + rT_0 - \frac{\gamma}{\beta}\ln T_0. \quad \text{(S3D.6)}$$

Integrating (S3D.6) from $t = 0$ to $t = t_{Vpeak}$ and using (S3C.6) we get

$$\int_0^{t_{Vpeak}} T dt = \frac{1}{r\beta}\ln\frac{\gamma}{r\beta T_0} + \frac{\gamma}{r\beta}\int_0^{t_{Vpeak}} \ln T(t) dt + \frac{1}{r}\left(V_0 + rT_0 - \frac{\gamma}{\beta}\ln T_0\right)t_{Vpeak}. \quad \text{(S3D.7)}$$

For $t \in [0, t_{Vpeak}]$ we approximate $\ln T(t)$ by $\ln T_0$, and thus $\int_0^{t_{Vpeak}} \ln T(t) dt \approx t_{Vpeak}\ln T_0$. Substituting into equation (S3D.7), and then (S3D.7) into (S3D.4), after some algebra we get

$$t_{Vpeak(TV)} \approx \frac{1}{\gamma - \beta\left(V_0 + rT_0\right)}\ln\frac{\gamma V_0}{r\beta T_0 V\left(t_{Vpeak}\right)_{(TV)}}, \quad \text{(S3D.8)}$$

where $V\left(t_{Vpeak}\right)_{(TV)}$ is given by (S3C.8).

It should be noted that in equation (S3D.7), as $\int_0^{t_{Vpeak}} \ln T(t) dt$ is multiplied by $\gamma / r\beta$ which is large, the approximation $\int_0^{t_{Vpeak}} \ln T(t) dt = t_{Vpeak}\ln T_0$ introduces a non-negligible degree of inaccuracy.

S3E Duration of infection

TV model

We define the duration of the infection as the time the virus in the body is above the detection limit (this was set to be equal to 0.7 $TCID_{50}$/ml, which is 0.05 $TCID_{50}$/ml below the smallest measurement value in the two datasets considered). From equation (S3D.6), at time t_{d_2}, which is the time at which the viral load reaches the detection limit during the decay phase, the viral load is given by

$$V(t_{d_2})_{(TV)} \quad = \quad -rT(t_{d_2}) + \frac{\gamma}{\beta}\ln T(t_{d_2}) + V_{t_{d1}} + rT_{t_{d1}} - \frac{\gamma}{\beta}\ln T_{t_{d1}}, \tag{S3E.1}$$

where V_0 and T_0 in (S3D.6) have been substituted by $V_{t_{d1}}$ and $T_{t_{d1}}$, respectively.

Dividing both sides of equation (5) by $-\beta VT$, substituting (S3E.1) into this and integrating from $t = t_{d_1}$ to $t = t_{d_2}$ we derive the infection duration $t_d = t_{d_2} - t_{d_1}$:

$$t_d \quad = \quad -\frac{1}{\beta}\int_{T(t_{d_1})}^{T(t_{d_2})} \frac{dT}{\left(-rT + \frac{\gamma}{\beta}\ln T + V_{t_{d1}} + rT_{t_{d1}} - \frac{\gamma}{\beta}\ln T_{t_{d1}}\right)T}, \tag{S3E.2}$$

where $T(t_{d_2})$ is given by (S3B.6).

To get an approximation of the infection duration we follow the following process.
Dividing (6) by V and integrating from $t = t_{d_1}$ to $t = t_{d_2}$ we get

$$\int_{t_{d_1}}^{t_{d_2}} T dt \quad = \quad \frac{1}{r\beta}\left(\ln V\left(t_{d_2}\right) - \ln V\left(t_{d_1}\right) + \gamma\left(t_{d_2} - t_{d_1}\right)\right) \tag{S3E.3}$$

$$\overset{t_d = t_{d_2} - t_{d_1}}{=>} \quad t_d \quad = \quad \frac{r\beta}{\gamma}\int_{t_{d_1}}^{t_{d_2}} T dt + \frac{1}{\gamma}\left(\ln V\left(t_{d_1}\right) - \ln V\left(t_{d_2}\right)\right). \tag{S3E.4}$$

Assume that

$$\int_{t_{d_1}}^{t_{d_2}} T dt \quad = \quad \int_{t_{d_1}}^{t_{Vpeak}} T(t)dt + \left(t_{d_2} - t_{Vpeak}\right)T_{t_{d_2}(TV)}. \tag{S3E.5}$$

Integrating (S3D.6) from $t = t_{d_1}$ to $t = t_{Vpeak}$ and using (S3C.6) we get

$$\int_{t_{d_1}}^{t_{Vpeak}} T(t)dt \quad = \quad -\frac{1}{r\beta}\ln\frac{r\beta T_{t_{d_1}}}{\gamma} + \frac{\gamma}{r\beta}\int_{t_{d_1}}^{t_{Vpeak}} \ln T(t)dt + \frac{1}{r}\left(V_{t_{d_1}} + rT_{t_{d_1}} - \frac{\gamma}{\beta}\ln T_{t_{d_1}}\right)\left(t_{Vpeak} - t_{d_1}\right). \tag{S3E.6}$$

Assume that

$$\int_{t_{d_1}}^{t_{Vpeak}} \ln T(t)dt \quad = \quad \left(t_{Vpeak} - t_{d_1}\right)\ln T_{t_{d_1}}. \tag{S3E.7}$$

Substituting (S3E.7) into (S3E.6) we get

$$\int_{t_{d_1}}^{t_{Vpeak}} T(t)dt = -\frac{1}{r\beta}\ln\frac{r\beta T_{t_{d_1}}}{\gamma}+\frac{1}{r}\left(V_{t_{d_1}}+rT_{t_{d_1}}\right)\left(t_{Vpeak}-t_{d_1}\right). \quad \text{(S3E.8)}$$

Substituting (S3E.8) into (S3E.5) we get

$$\int_{t_{d_1}}^{t_{d_2}} Tdt = -\frac{1}{r\beta}\ln\frac{r\beta T_{t_{d_1}}}{\gamma}+\frac{1}{r}\left(V_{t_{d_1}}+rT_{t_{d_1}}\right)\left(t_{Vpeak}-t_{d_1}\right)+\left(t_{d_2}-t_{Vpeak}\right)T_{t_{d_2}(TV)}. \quad \text{(S3E.9)}$$

Substituting (S3E.9) into (S3E.4) we get

$$\begin{aligned} t_d &= \frac{1}{\gamma}\left(\ln\frac{\gamma}{r\beta}-\ln T(t_{d_1})\right)+\frac{\beta}{\gamma}\left(V_{t_{d_1}}+rT_{t_{d_1}}\right)\left(t_{Vpeak}-t_{d_1}\right) \\ &\quad +\frac{r\beta}{\gamma}\left(t_{d_2}-t_{Vpeak}\right)T_{t_{d_2}(TV)}+\frac{1}{\gamma}\left(\ln V(t_{d_1})-\ln V(t_{d_2})\right). \end{aligned} \quad \text{(S3E.10)}$$

Assuming that $t_{d_1}=0$ we get

$$t_{d\,App} = \frac{1}{\gamma-r\beta T_{t_{d_2}(TV)}}\left(\ln\frac{\gamma}{r\beta T_0}+r\beta t_{Vpeak(TV)}\left(\frac{V_0}{r}+T_0-T_{t_{d_2}(TV)}\right)-\ln\frac{V_{t_{d_2}}}{V_0}\right). \quad \text{(S3E.11)}$$

where $T_{t_{d_2}(TV)}$ is given explicitly by (S3B.6), $t_{Vpeak(TV)}$ by (S3D.8) and $V_{t_{d_2}}$ by (S3E.1) (with $t_{d_1}=0$).

S3F Generation time

Under the assumption that infectiousness is proportional to viral shedding and the population is homogeneous, the average generation time (at the population level) during the time the virus is detectable is given by

$$T_g = \frac{\int_{t_{d_1}}^{t_{d_2}} tV(t)dt}{A_V}, \quad \text{(S3F.1)}$$

where A_V is the area under the viral load curve; T_g is the mean of the infectiousness distribution [7-9].

TIV model

Assume that $t_{d_1}\approx 0$.

Substituting (S3A.2) into (3), multiplying by t and integrating from $t = 0$ to $t = t_{d_2}$ we get

$$\int_0^{t_{d_2}} tV(t)dt = -\frac{p}{c\delta_I}\int_0^{t_{d_2}} tdT - \frac{p}{c\delta_I}\int_0^{t_{d_2}} tdI - \frac{1}{c}\int_0^{t_{d_2}} tdV \,. \tag{S3F.2}$$

Using integration by parts we get

$$\int_0^{t_{d_2}} tdT = t_{d_2} T_{t_{d_2}(TIV)} - \int_0^{t_{d_2}} T(t)dt \,. \tag{S3F.3}$$

$$\int_0^{t_{d_2}} tdI = -\int_0^{t_{d_2}} I(t)dt = \frac{1}{\delta_I}\left(T_{t_{d_2}(TIV)} - T_0 - I_0\right). \tag{S3F.4}$$

$$\int_0^{t_{d_2}} tdV = -\int_0^{t_{d_2}} V(t)dt = -A_{V(TIV)} \,. \tag{S3F.5}$$

$T_{t_{d_2}(TIV)}$ is given by (S3B.2).

Substituting (S3F.3)-(S3F.5) into (S3F.2) and using (S3A.4) we get

$$\int_0^{t_{d_2}} tV(t)dt = \frac{p}{c\delta_I}\int_0^{t_{d_2}} T(t)dt - \frac{p}{c\delta_I} t_{d_2} T_0 - \frac{1}{c}\left(t_{d_2} + \frac{1}{\delta_I}\right)V_0 - \frac{p}{c\delta_I} t_{d_2} I_0 + \left(t_{d_2} + \frac{1}{\delta_I} + \frac{1}{c}\right)A_{V(TIV)} \,. \tag{S3F.6}$$

The area under the curve of target cells is given by

$$\int_0^{t_{d_2}} T(t)dt = \int_0^{t^*} T(t)dt + \left(t_{d_2} - t^*\right)T_{t_{d_2}(TIV)} \,, \tag{S3F.7}$$

where t^* is the time required for the target cells to reach the state $T_{t_{d_2}}$ $\left(t^* < t_{d_2}\right)$. We assume that the target cells remain almost constant before their rapid decline to $T_{t_{d_2}}$, that is $\int_0^{t^*} T(t)dt = T_0 t^*$. Although this approximation introduce a degree of inaccuracy, this is small due to the division of this term by $A_{V(TIV)}$. Substituting into (S3F.6) and using (S3F.1) we derive

$$T_g = \frac{-\frac{1}{c}\left(\frac{1}{\delta_I} + t^*\right)V_0 - \frac{p}{c\delta_I} t^* I_0}{A_{V(TIV)}} + t^* + \frac{1}{\delta_I} + \frac{1}{c} \approx t^* + \frac{1}{\delta_I} + \frac{1}{c} \,. \tag{S3F.8}$$

A good approximation of t^* is $t_{Vpeak(TIV)}$ given by (S3D.3).

Increasing the infection rate, the viral production rate, the initial number of target cells or the initial viral load yields lower T_g values. In this case, the generation time approaches $1/\delta + 1/c$, which is the average life span of an infected cell and the average life span of a free virion (see also [10]).

TV model

Multiplying (6) by t, using (5) and integrating from $t = t_{d_1}$ to $t = t_{d_2}$ we get

$$\int_{t_{d_1}}^{t_{d_2}} tVdt \quad = \quad -\frac{r}{\gamma}\int_{t_{d_1}}^{t_{d_2}} tdT - \frac{1}{\gamma}\int_{t_{d_1}}^{t_{d_2}} tdV \,. \tag{S3F.9}$$

Using integration by parts we get

$$\int_{t_{d_1}}^{t_{d_2}} t\frac{dV}{dt}dt \quad = \quad t_{d_2}V(t_{d_2}) - t_{d_1}V(t_{d_1}) - A_{V(TV)} \,. \tag{S3F.10}$$

$$\int_{t_{d_1}}^{t_{d_2}} tdT \quad = \quad t_{d_2}T\left(t_{d_2}\right) - t_{d_1}T\left(t_{d_1}\right) - \int_{t_{d_1}}^{t_{d_2}} Tdt \,. \tag{S3F.11}$$

Substituting (S3F.10) and (S3F.11) into (S3F.9) and then into (6) we get

$$T_{g(TV)} \quad = \quad \frac{-\frac{r}{\gamma}\left(t_{d_2}T\left(t_{d_2}\right) - t_{d_1}T\left(t_{d_1}\right) - \int_{t_{d_1}}^{t_{d_2}} Tdt\right) - \frac{1}{\gamma}\left(t_{d_2}V(t_{d_2}) - t_{d_1}V(t_{d_1}) - A_{V(TV)}\right)}{A_{V(TV)}} \,. \tag{S3F.12}$$

Substituting (S3E.3) into (S3F.12) we get

$$T_{g(TV)} \quad = \quad \frac{-\frac{r}{\gamma}t_{d_2}T\left(t_{d_2}\right) + \frac{r}{\gamma}t_{d_1}T\left(t_{d_1}\right) + \frac{1}{\beta\gamma}\ln\frac{V\left(t_{d_2}\right)}{V\left(t_{d_1}\right)} - \frac{1}{\gamma}t_{d_2}V(t_{d_2}) + \frac{1}{\gamma}t_{d_1}V(t_{d_1}) + \frac{1}{\beta}t_{d_2} - \frac{1}{\beta}t_{d_1}}{A_{V(TV)}} + \frac{1}{\gamma} \,. \tag{S3F.13}$$

Assuming that $t_{d_1} \approx 0$ and using (S3A.20) and (S3E.1) we get

$$T_{g(TV)\text{App}} \quad = \quad \frac{t_{dApp}\left(\frac{1}{\beta} - \frac{1}{\gamma}V_0 - \frac{r}{\gamma}T_0 + A_{V(TV)}\right) + \frac{1}{\beta\gamma}\ln\frac{V_0 - \gamma A_{V(TV)} + rT_0\left(1 - e^{-\beta A_{V(TV)}}\right)}{V_0}}{A_{V(TV)}} + \frac{1}{\gamma} \,, \tag{S3F.14}$$

where t_{dApp} is the duration of the infection given by (S3E.11).

References

1. Corless RM, Gonnet GH, Hare DEG, Jeffrey DJ, Knuth DE. On the LambertW function. Adv Comput Math. 1996;5(1):329-59.

2. Luo S, Reed M, Mattingly JC, Koelle K. The impact of host immune status on the within-host and population dynamics of antigenic immune escape. Journal of the Royal Society Interface. 2012;9(75):2603-13.

3. Diekmann O, Heesterbeek JAP. Mathematical Epidemiology of Infectious Diseases: Model Building, Analysis and Interpretation: Wiley; 2000.
4. Diekmann O, Heesterbeek H, Britton T. Mathematical Tools for Understanding Infectious Disease Dynamics: Princeton University Press; 2013.
5. Nowak MA, Lloyd AL, Vasquez GM, Wiltrout TA, Wahl LM, Bischofberger N, et al. Viral dynamics of primary viremia and antiretroviral therapy in simian immunodeficiency virus infection. Journal of virology. 1997;71(10):7518-25.
6. Lee HY, Topham DJ, Park SY, Hollenbaugh J, Treanor J, Mosmann TR, et al. Simulation and prediction of the adaptive immune response to influenza A virus infection. Journal of virology. 2009;83(14):7151-65.
7. Fraser C, Riley S, Anderson RM, Ferguson NM. Factors that make an infectious disease outbreak controllable. Proceedings of the National Academy of Sciences of the United States of America. 2004;101(16):6146-51.
8. Carrat F, Vergu E, Ferguson NM, Lemaitre M, Cauchemez S, Leach S, et al. Time lines of infection and disease in human influenza: a review of volunteer challenge studies. American journal of epidemiology. 2008;167(7):775-85.
9. Canini L, Carrat F. Population modeling of influenza A/H1N1 virus kinetics and symptom dynamics. Journal of virology. 2011;85(6):2764-70.
10. Perelson AS, Neumann AU, Markowitz M, Leonard JM, Ho DD. HIV-1 dynamics in vivo: virion clearance rate, infected cell life-span, and viral generation time. Science. 1996;271(5255):1582-6.
