# Supplementary material for: Understanding the within-host dynamics of influenza A virus: from theory to clinical implications
Source: J R Soc Interface. 2016 Jun;13(119):20160289. doi: 10.1098/rsif.2016.0289 (PMC4938090; doi:10.1098/rsif.2016.0289)
Supplement: Supplementary material S4 [file rsif20160289supp4.pdf]

Supplementary material S4. Figures

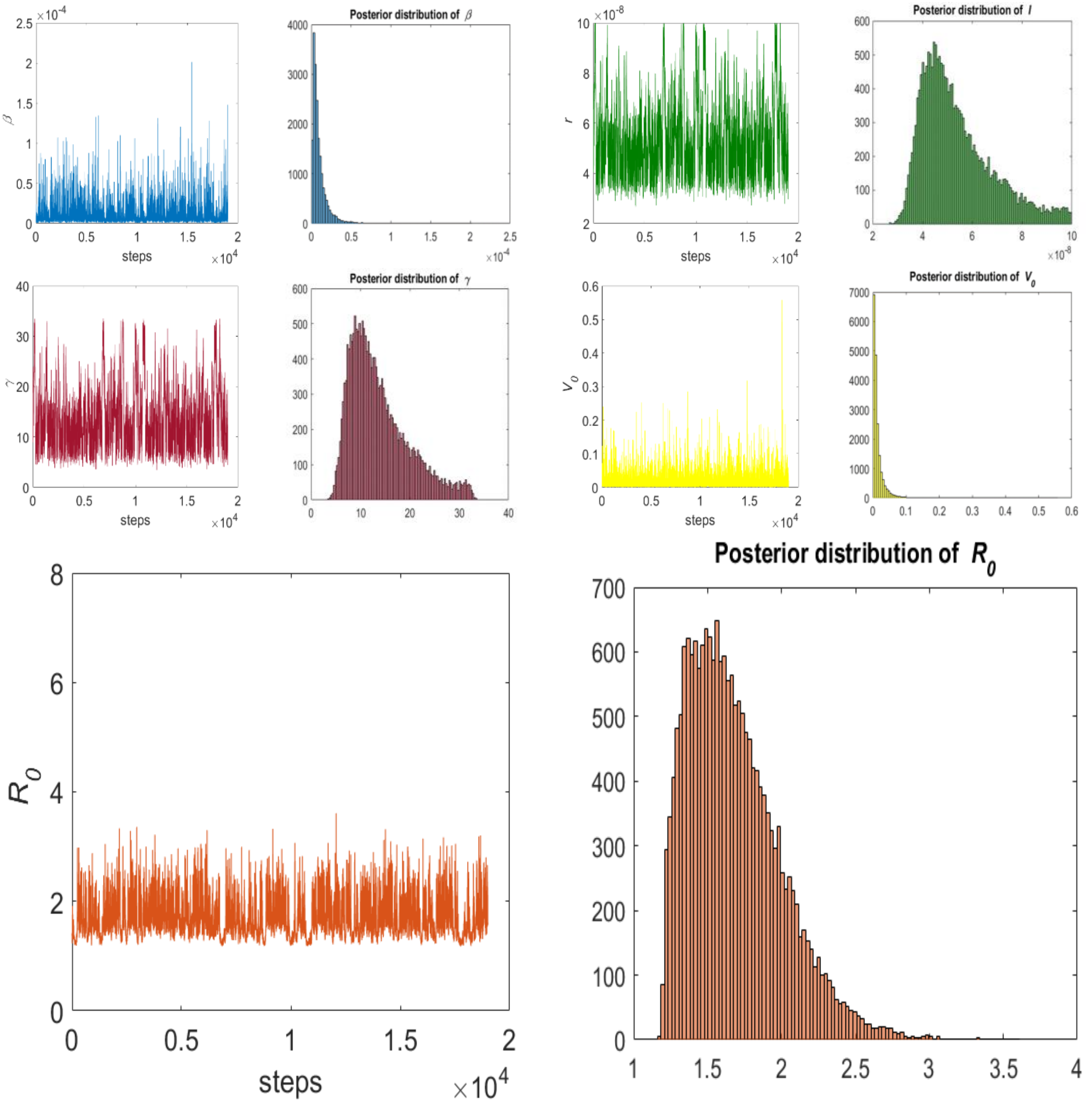

Figure S4.1: Trace plots show the values that the parameters β, l, γ, V_0 and R_0 took during the runtime of the chain for patient 5 in the oseltamivir trial. Histograms show the posterior distribution of each of the parameters.

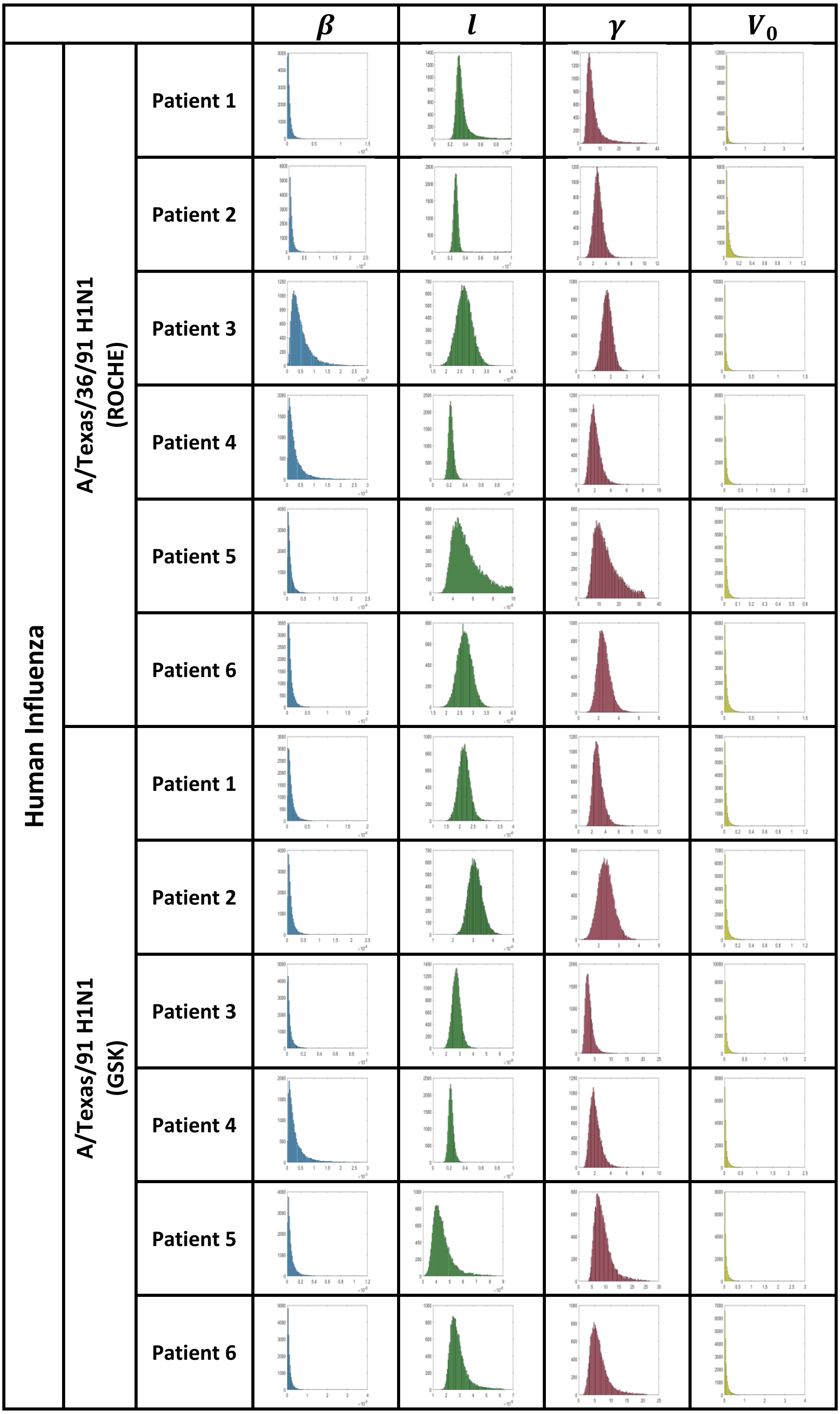

Figure S4.2: Posterior distributions of each of the unknown parameters.

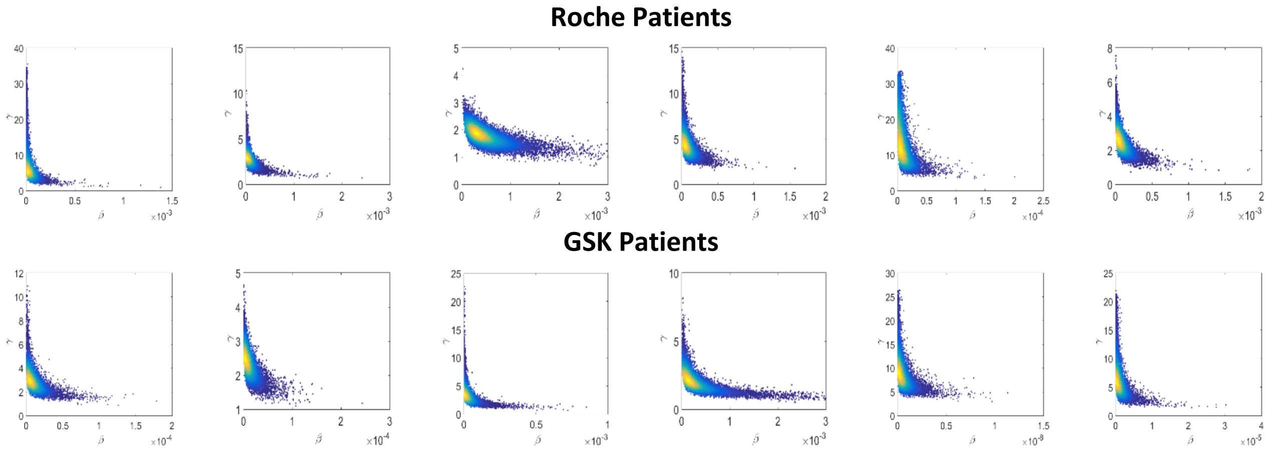

Figure S4.3: Scatter plots of points in (β, γ) plane. Points are coloured according to their likelihood value, with the blue colour indicating the least likely values and the yellow the most likely values.

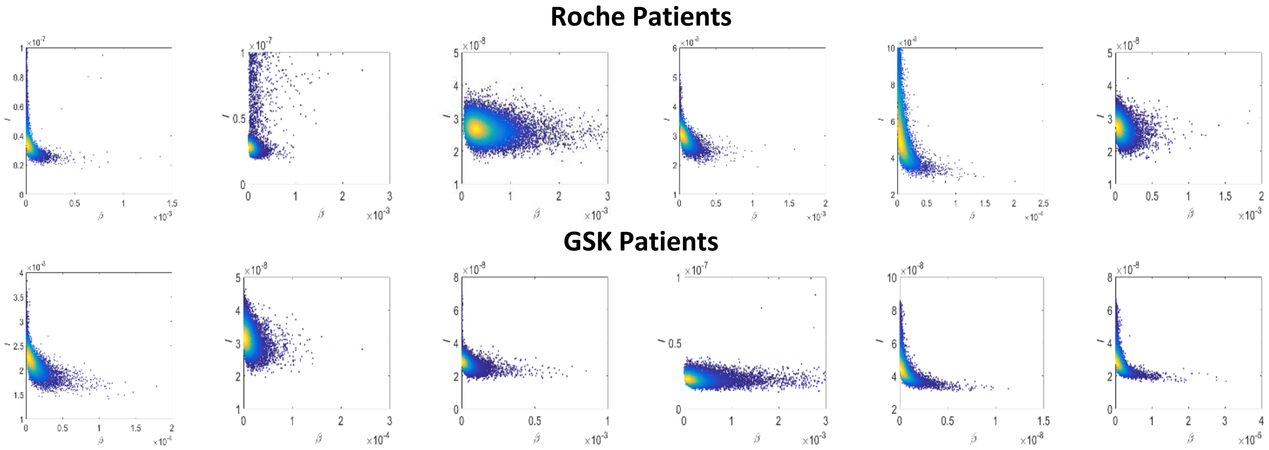

Figure S4.4: Scatter plots of points in (β, l) plane. Points are coloured according to their likelihood value, with the blue colour indicating the least likely values and the yellow the most likely values.

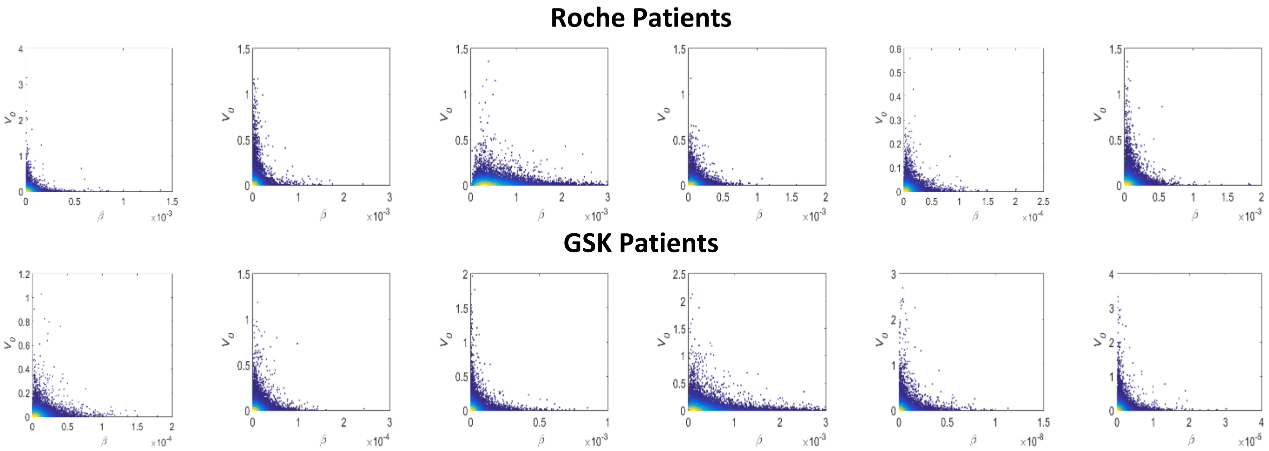

Figure S4.5: Scatter plots of points in (β, V_0) plane. Points are coloured according to their likelihood value, with the blue colour indicating the least likely values and the yellow the most likely values.

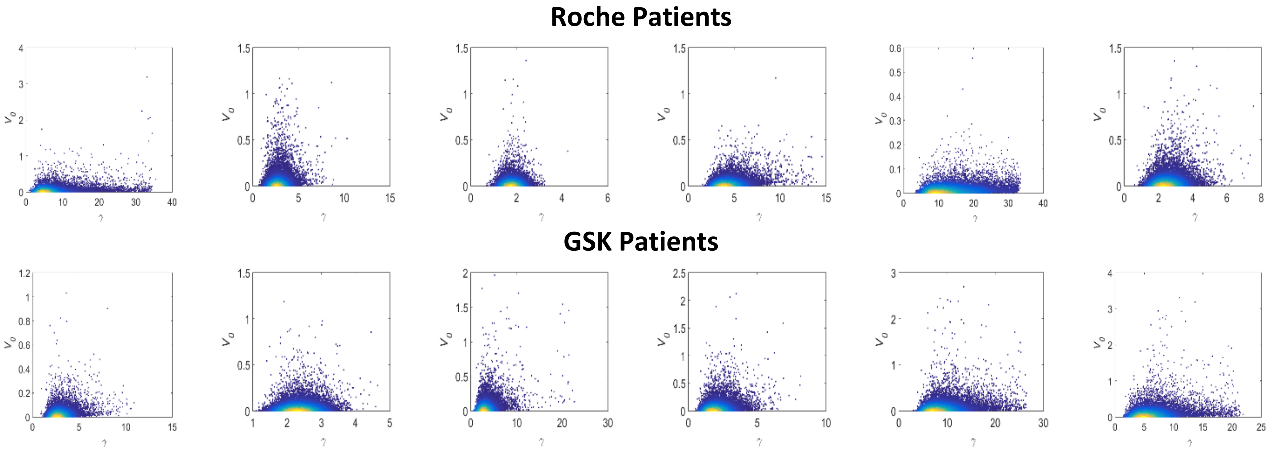

Figure S4.6: Scatter plots of points in (γ, V_0) plane. Points are coloured according to their likelihood value, with the blue colour indicating the least likely values and the yellow the most likely values.

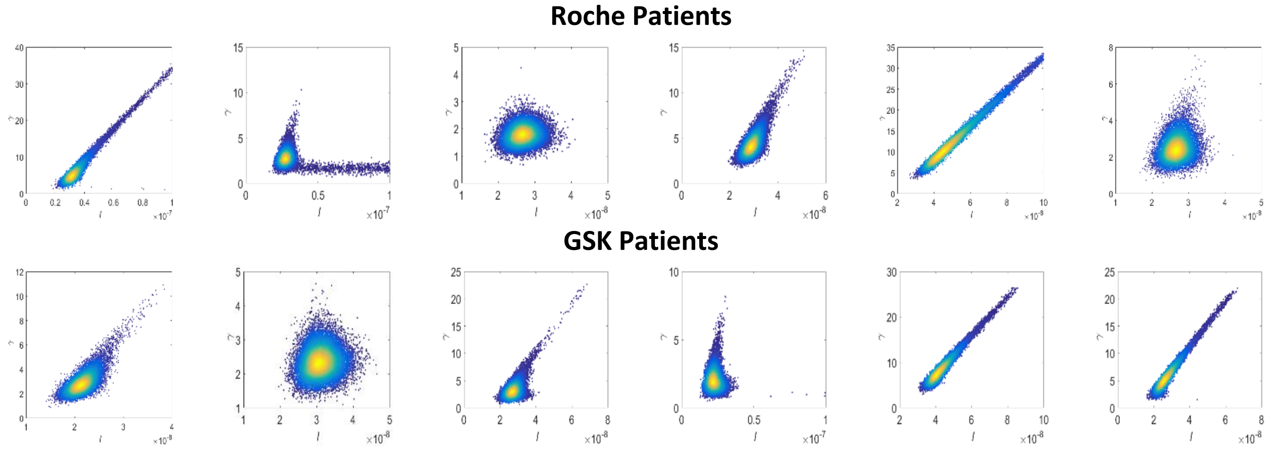

Figure S4.7: Scatter plots of points in (l, γ) plane. Points are coloured according to their likelihood value, with the blue colour indicating the least likely values and the yellow the most likely values.

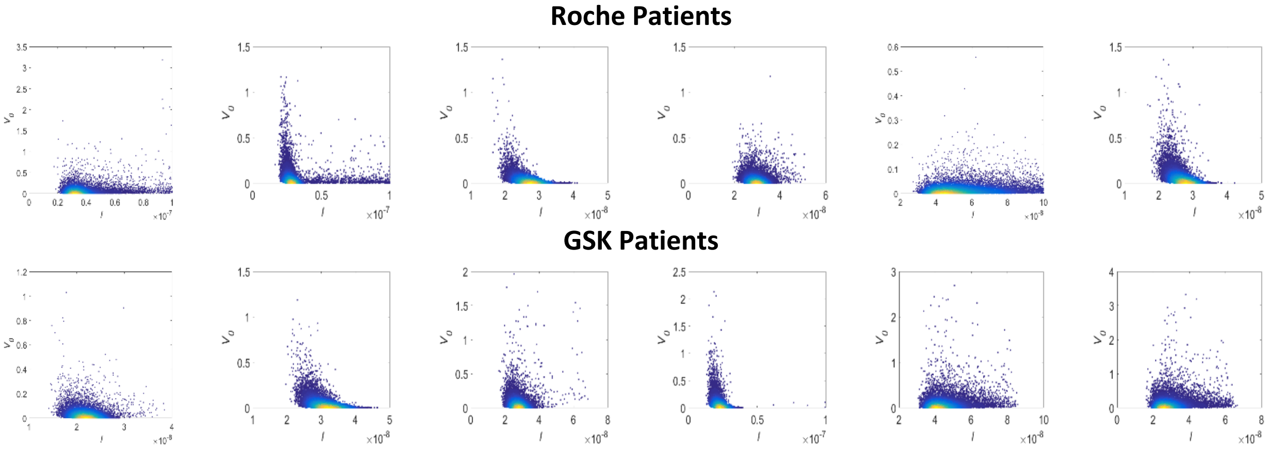

Figure S4.8: Scatter plots of points in (l, V_0) plane. Points are coloured according to their likelihood value, with the blue colour indicating the least likely values and the yellow the most likely values.
