# Supplementary material for: Understanding the within-host dynamics of influenza A virus: from theory to clinical implications
Source: J R Soc Interface. 2016 Jun;13(119):20160289. doi: 10.1098/rsif.2016.0289 (PMC4938090; doi:10.1098/rsif.2016.0289)
Supplement: Supplementary material S5 [file rsif20160289supp5.pdf]

Supplementary material S5. Estimates of model parameters

Table S5. Estimated posterior medians of individual parameters and the corresponding 95% credible intervals.

			Estimates of the parameters in the TV model				
			**Initial viral load, V_0 ($TCID_{50}$/ml)**	**Initial number of target cells, T_0 (cells)**	**Infection rate, β [$(TCID_{50}/ml)^{-1}$ x day^{-1}]**	**Parameter l (1/day)**	**Clearance rate of virus, γ (1/day)**
**Human Influenza**	**A/Texas/36/91 H1N1 (ROCHE)**	**Patient 1**	0.023 [0.002 0.27]	4 × 10^8 (fixed)	2.67 x 10^{-5} [3.56 x 10^{-6} 1.58 x 10^{-4}]	3.32 x 10^{-8} [2.55 x 10^{-8} 6.96 x 10^{-8}]	5.59 [2.74 21.68]
		**Patient 2**	0.023 [0.002 0.26]		5.97 x 10^{-5} [1.30 x 10^{-5} 3.01 x 10^{-4}]	2.77 x 10^{-8} [2.26 x 10^{-8} 6.24 x 10^{-8}]	2.73 [1.55 4.56]
		**Patient 3**	0.014 [0.001 0.14]		3.99 x 10^{-4} [1.01 x 10^{-4} 0.001]	2.67 x 10^{-8} [2.13 x 10^{-8} 3.29 x 10^{-8}]	1.78 [1.22 2.44]
		**Patient 4**	0.0175 [0.002 0.16]		6.16 x 10^{-5} [1.36 x 10^{-5} 2.76 x 10^{-4}]	2.94 x 10^{-8} [2.42 x 10^{-8} 3.58 x 10^{-8}]	4.18 [2.65 7.41]
		**Patient 5**	0.008 [9.53 x 10^{-4} 0.066]		6.62 x 10^{-6} [1.22 x 10^{-6} 3.71 x 10^{-5}]	5.04 x 10^{-8} [3.48 x 10^{-8} 9.21 x 10^{-8}]	12.34 [5.91 29.56]
		**Patient 6**	0.025 [0.002 0.25]		6.48 x 10^{-5} [1.39 x 10^{-5} 3.13 x 10^{-4}]	2.68 x 10^{-8} [2.17 x 10^{-8} 3.23 x 10^{-8}]	2.44 [1.47 3.99]
	**A/Texas/91 H1N1 (GSK)**	**Patient 1**	0.016 [0.002 0.13]		7.19 x 10^{-6} [1.32 x 10^{-6} 3.96 x 10^{-5}]	2.16 x 10^{-8} [1.76 x 10^{-8} 2.64 x 10^{-8}]	2.82 [1.73 5.07]
		**Patient 2**	0.02 [0.0017 0.2]		7.84 x 10^{-6} [1.42 x 10^{-6} 4.34 x 10^{-5}]	3.09 x 10^{-8} [2.50 x 10^{-8} 3.83 x 10^{-8}]	2.33 [1.64 3.20]
		**Patient 3**	0.025 [0.002 0.25]		2.06 x 10^{-5} [2.57 x 10^{-6} 1.60 x 10^{-4}]	2.75 x 10^{-8} [2.17 x 10^{-8} 3.48 x 10^{-8}]	3.02 [1.60 6.76]
		**Patient 4**	0.032 [0.003 0.32]		1.99 x 10^{-4} [2.90 x 10^{-5} 0.001]	2.23 x 10^{-8} [1.71 x 10^{-8} 3.01 x 10^{-8}]	1.96 [1.07 3.81]
		**Patient 5**	0.035 [0.003 0.39]		3.61 x 10^{-10} [4.75 x 10^{-11} 2.56 x 10^{-9}]	4.27 x 10^{-8} [3.51 x 10^{-8} 6.56 x 10^{-8}]	8.32 [4.93 18.27]
		**Patient 6**	0.05 [0.005 0.5]		7.27 x 10^{-7} [1.21 x 10^{-7} 4.90 x 10^{-6}]	2.73 x 10^{-8} [2.06 x 10^{-8} 4.82 x 10^{-8}]	5.70 [2.70 14.72]
